# Future global productivity will be affected by plant trait response to climate

**DOI:** 10.1038/s41598-018-21172-9

**Published:** 2018-02-12

**Authors:** Nima Madani, John S. Kimball, Ashley P. Ballantyne, David L. R. Affleck, Peter M. van Bodegom, Peter B. Reich, Jens Kattge, Anna Sala, Mona Nazeri, Matthew O. Jones, Maosheng Zhao, Steven W. Running

**Affiliations:** 1 Numerical Terradynamic Simulation Group, W.A. Franke College of Forestry & Conservation, University of Montana, Missoula, MT 59812 USA; 2 Department of Ecosystem and Conservation Sciences, W.A. Franke College of Forestry & Conservation, University of Montana, Missoula, Montana 59812 USA; 3 Department of Forest Management, W.A. Franke College of Forestry & Conservation, University of Montana, 32 Campus Drive, Missoula, MT 59812 USA; 4 Institute of Environmental Sciences (CML), University Leiden, 2333CC Leiden, The Netherlands; 5 Department of Forest Resources, University of Minnesota, 1530 Cleveland Avenue North, St. Paul, Minnesota 55108 USA; 6 Hawkesbury Institute for the Environment, Western Sydney University, Penrith, 2753 NSW Australia; 7 Max-Planck-Institute for Biogeochemistry, 07745 Jena, Germany; 8 German Centre for Integrative Biodiversity Research (iDiv) Halle-Jena-Leipzig, 04103 Leipzig, Germany; 9 Division of Biological Sciences, University of Montana, Missoula, MT 59812 USA; 10 Department of Wildlife, Fisheries and Aquaculture, Mississippi State University, MS, 39762 USA; 11 Department of Geographical Sciences, University of Maryland, College Park, Maryland 20742 USA

## Abstract

Plant traits are both responsive to local climate and strong predictors of primary productivity. We hypothesized that future climate change might promote a shift in global plant traits resulting in changes in Gross Primary Productivity (GPP). We characterized the relationship between key plant traits, namely Specific Leaf Area (SLA), height, and seed mass, and local climate and primary productivity. We found that by 2070, tropical and arid ecosystems will be more suitable for plants with relatively lower canopy height, SLA and seed mass, while far northern latitudes will favor woody and taller plants than at present. Using a network of tower eddy covariance CO2 flux measurements and the extrapolated plant trait maps, we estimated the global distribution of annual GPP under current and projected future plant community distribution. We predict that annual GPP in northern biomes (≥45 °N) will increase by 31% (+8.1 ± 0.5 Pg C), but this will be offset by a 17.9% GPP decline in the tropics (−11.8 ± 0.84 Pg C). These findings suggest that regional climate changes will affect plant trait distributions, which may in turn affect global productivity patterns.

## Introduction

Climate change is expected to significantly influence global species distributions in the next decades^1,2^, which raises the question of how these changes may affect dominant plant community traits and ecosystem productivity. The response of species to climate change can vary from extinction to resilience^3^. However, plant species may also adapt to climate change by altering their physical traits^3^ or by relocating to regions with more suitable environmental conditions^4,5^. Increases in shrub dominance in the tundra^6^ and declines in taller, larger diameter trees in California in the last century, inducing a shift toward oak dominance over historic pine dominance^7^, provide recent examples of such changes.

Temperature, water supply and solar radiation are primary climatic factors constraining ecosystem productivity at global scales^8,9^ such that each or a combination of these factors limits vegetation growth within global biomes defined by species with distinctive traits and/or life history strategies. From the ecosystem process perspective, vegetation productivity has increased in recent decades^8,10^. Plant productivity may be enhanced through direct fertilization effects from increasing atmospheric CO_2_ concentrations^11,12^. However, concomitant changes in temperature and rainfall can also alter productivity by extending the growing season in cold regions, while limiting productivity in warmer and drier regions^13^. A key, unresolved question is how changes in precipitation and temperature will affect species functional traits and what impact changes in traits and plant communities will have on patterns of global productivity.

Plant traits have been shown to provide important information about ecosystem structure and productivity^14^. Plants have distinctive strategies that manifest as functional traits adapted to local habitats and environmental conditions^15,16^, and yet trade-offs among functional traits can reveal and influence ecosystem processes^14,17–19^. Leaf traits such as leaf nitrogen content (N) and SLA (the ratio of leaf area per unit dry mass, m^2^ kg^−1^) influence canopy photosynthetic capacity^20^ and have been shown to improve understanding of key ecosystem processes such as GPP^21,22^. SLA, vegetation height, and seed mass are among the most widely used plant traits in ecological studies and can explain species distributions^16,23,24^. The leaf-height-seed (LHS) relationship was proposed to help explain species co-existence strategies: while height and seed mass reflect capabilities to cope with environmental disturbance, SLA distinguishes between competitors and stress-tolerators^16^. The LHS relationship is also related to ecosystem function. Leaves with higher SLA generally have higher nutrient (N) concentrations^15,17,19^, leading to higher carbon assimilation^22^ and respiration^25^. Species with higher seed mass are generally found in more productive regions^26–28^ and can tolerate a higher degree of stresses, while species with lower seed mass need relatively less energy for seed production^29^. Taller trees tend to have greater access to light, deploy more canopy leaf area and have higher leaf nitrogen content^30,31^.

Despite the influence of morphological plant traits on ecosystem properties and function, their role in global ecosystem process models is often neglected or not properly captured^21^. Many global models use generalized plant functional type (PFT) categories to explain differences in ecosystem function^22^. While these functional types are distinguishable using physical plant traits^23^, large variability in ecosystem function within individual PFT classes^22,32^ suggests that such broad categories are insufficient in modeling ecosystem processes such as productivity. Such uncertainty may contribute to the large range of estimated global annual GPP (106–175 Pg C yr^−1^) from different models^33–36^. However, recent attempts to map global plant traits^23^, the effect of future climate conditions on community trait patterns^37^, and incorporation of plant trait information into earth system models^38,39^ have improved our understanding of climate impacts on plant community patterns and ecosystem productivity.

In this research we characterize the relationships between bioclimatic variables and plant traits using a global plant trait database (TRY)^40^. Specifically, we analyze relationships between gridded bioclimatic factors related to precipitation and temperature, and selected key dominant community plant traits. Via analyses of annual GPP derived from 164 globally distributed carbon flux towers, we show that ecosystem productivity is significantly related to the plant trait observations. We then use selected bioclimatic variables^41^ from 17 global Earth System Models (ESMs) of the Intergovernmental Panel on Climate Change (IPCC) Coupled Model Intercomparison Project Phase 5 (CMIP5) based on the Representative Concentration Pathway (RCP) 8.5^42^ for the year 2070 to predict changes in key plant traits under projected future climate change. We find that changes in ecosystem suitability favor plants with certain functional traits, and that projected climate change will impact both productivity and underlying community dominant functional traits.

## Results and Discussion

**Figure 1:**
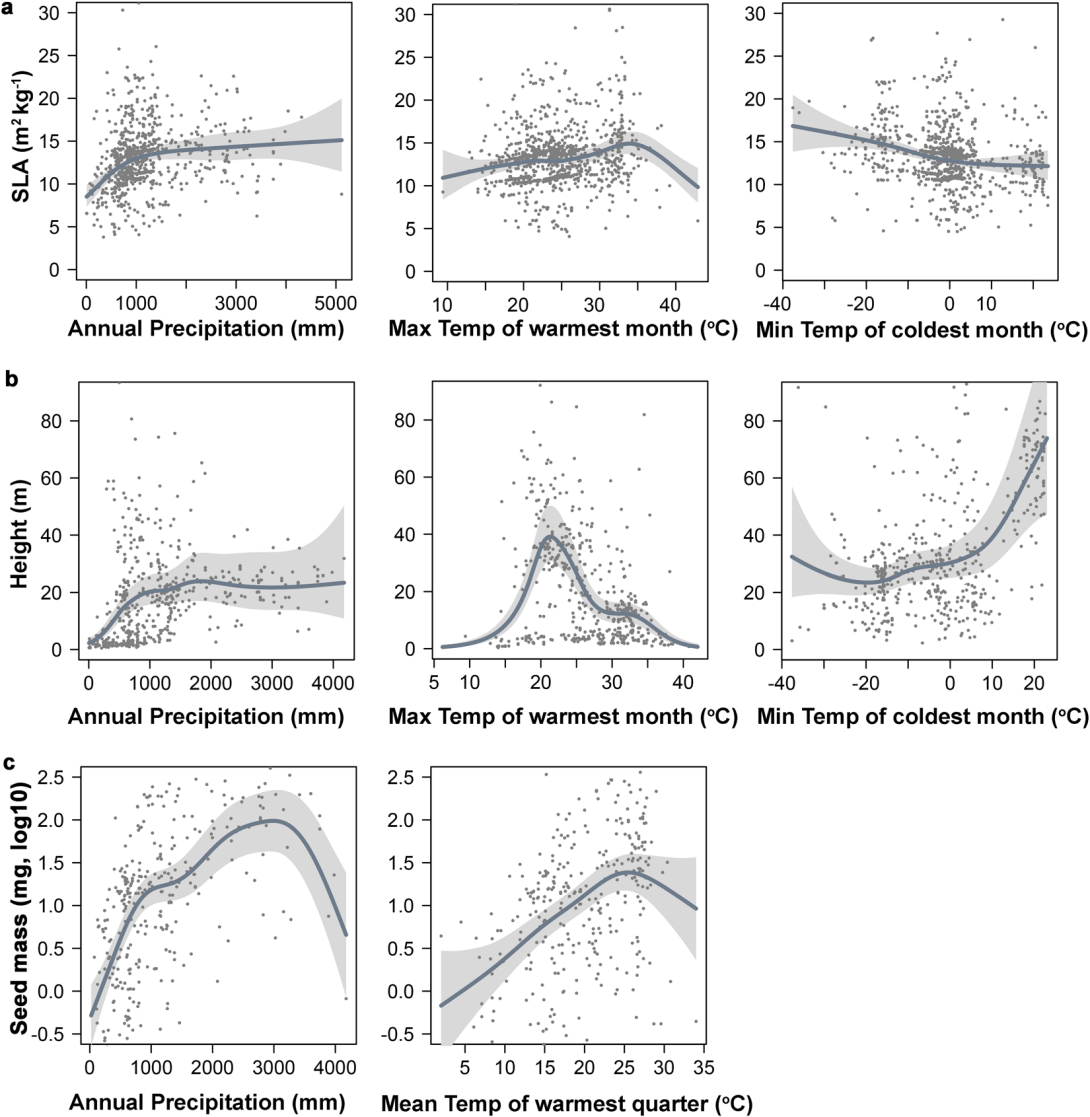
The estimated global relationships between the selected key plant trait and best performing climatic predictor variables. Smoothed functions were determined from fitted generalized additive models describing relationships between selected climate drivers and key global plant traits, including SLA (**a**) canopy height (**b**) and seed mass (**c**). The models were developed from climate variables and global plant trait observations including 1178, 329 and 520 data points for SLA, seed mass, and height, respectively. Shaded areas denote 95% confidence intervals, while gray dots represent partial residuals.

We used a Generalized Additive Model (GAM)^43^ to explain variability in species trait observations (Figure S2) relative to selected bioclimatic variables. The bioclimatic variables selected are based on stepwise variable selection and the best performance [Akaike’s Information Criterion (AIC)^44^ and lowest Root Mean Squared Errors (RMSE)] indicated from a leave-one-out cross validation analysis. Among the 19 available climatic variables analyzed from the WorldClim database^41^, SLA is mainly explained by annual average precipitation, maximum temperature of the warmest month and minimum temperature of the coldest month. Based on covariate analysis of the global GAM, SLA is proportional to precipitation (P) except for moist climates (P > ~1000 mm yr^−1^) where SLA is relatively insensitive to further P increases. SLA increases with maximum temperature of the warmest month, except for regions with warmer temperatures exceeding 35 degrees Celsius, which includes arid environments. However, SLA is inversely proportional to the minimum temperature of the coldest month. For example, tundra vegetation in arctic regions with cold winters shows higher SLA than temperate evergreen forests (Fig. 1).

Plant height increases with annual precipitation and levels off under wetter conditions above 2000 mm yr^−1^. Plant height also increases with the maximum temperature of the warmest month, approaching greatest height near 22 °C (tropical regions) and decreasing in areas with very warm temperatures (above 25 °C). Trees also inhabit boreal forests and other areas with cold winters, but canopy height generally increases as the minimum temperature of the coldest month rises (Fig. 1b). Seed mass is most responsive to annual precipitation and the mean temperature of the warmest annual quarter. The log10 of seed mass increases from low to moderate precipitation levels, but declines under higher rainfall amounts (exceeding ~2700 mm yr^−1^). The mean temperature of the warmest quarter, which generally represents the growing season, has a positive relationship with seed mass except for regions with very warm summer temperatures (exceeding ~25 °C) that are associated with lower seed mass plants (Fig. 1c).

**Figure 2:**
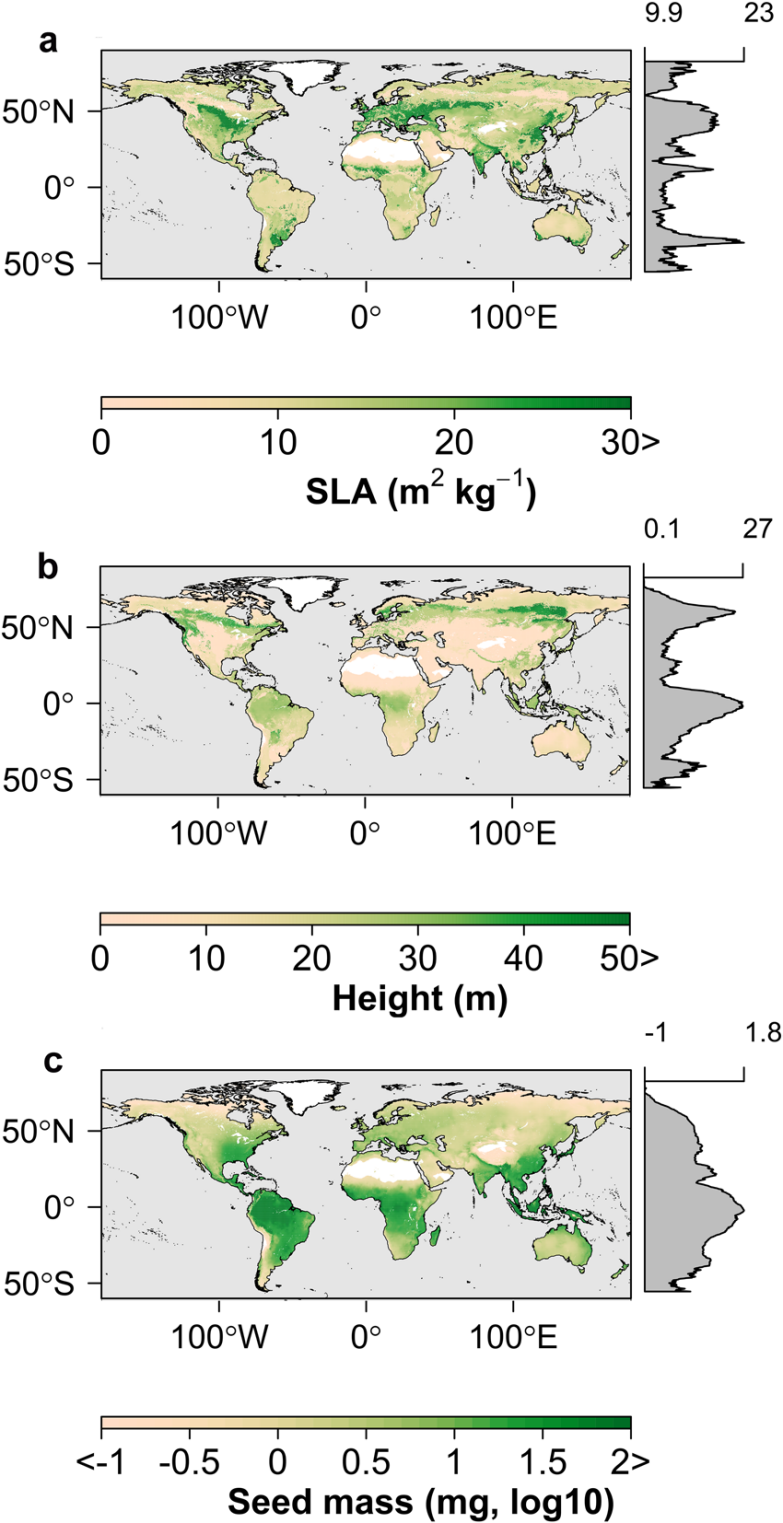
Global distribution of the estimated key plant traits. The global distribution of key plant traits (**a)** Specific Leaf Area (SLA), (**b)** Height, and (**c)** Seed Mass (SM)) represent dominant overstory condition derived from global plant trait observations^40^ and gridded climate variables^41^. Gray margins show latitudinal averages for each trait. The figure was created using the rasterVis library^81^ in R^77^.

The GAM results explain 68.2%, 66.2% and 45.5% of the variance among the SLA, height, and seed mass observations at the global scale (see Table S3 for regression coefficients of the smoothed functions used in the GAM). Using the global extrapolated plant trait information, we show global distribution of plant traits (Fig. 2). High stress areas (deserts and arid regions) are associated with plants with lower height, which is consistent with reported negative effects of low moisture availability on plant height^45^. These areas are also inhabited by plants with lower seed mass, which can promote seed dispersal^16^. Temperate evergreen trees that have high canopy height can enhance their water use efficiency by having low SLA, while also having a longer foliar life span and lower autotrophic respiration cost^46^. Plants in tropical biomes have relatively high values of the three traits, though this region is dominated by plants with greater height and seed mass.

**Figure 3:**
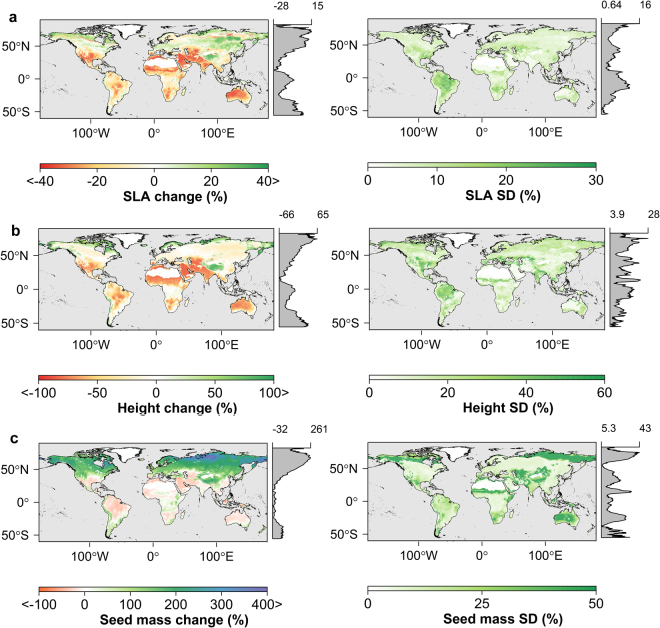
Potential changes in key plant traits as a result of projected near-term climate change. GAM projected changes in SLA (**a**), canopy height (**b**) and seed mass (**c**) under future (year 2070) climate conditions represented by the ensemble mean of 17 CMIP5 climate models and RCP 8.5 scenario relative to current conditions; the standard deviation in estimated plant traits derived from each of the 17 climate model outputs is also shown. Gray margins show latitudinal averages (%) for each trait. The figure was created using the rasterVis library^81^ in R^77^.

In order to evaluate how vegetation structure and ecosystem productivity may respond as a result of future climate change, we projected the GAM simulated plant traits using climate variables derived from 17 CMIP5 ESMs (Table S2) for the year 2070 based on the RCP8.5 greenhouse gas concentration trajectory^42^. GAM simulations were run on climate variables from each of the 17 ESMs and their ensemble mean. The standard deviation of the resulting GAM outputs derived from 17 ESM climate projection was used as a metric of uncertainty in the model projections. Based on the ensemble projection, boreal and arctic regions show the largest change in plant traits relative to other global biomes, with increases in SLA (+10–20%), canopy height (+20–30%), and seed mass (+25–200%) (Fig. 3). Our results also indicate that in the future, tropical regions may be inhabited by plants with an average 1 m^2^ kg^−1^ (−10%) lower SLA, 5.3 m (−12.5%) lower canopy height, and 0.15 mg (−9.1%) lower log10 seed mass relative to current conditions. These potential changes would not only affect large scale distributions of functional plant traits, but may also affect ecosystem productivity.

**Figure 4:**
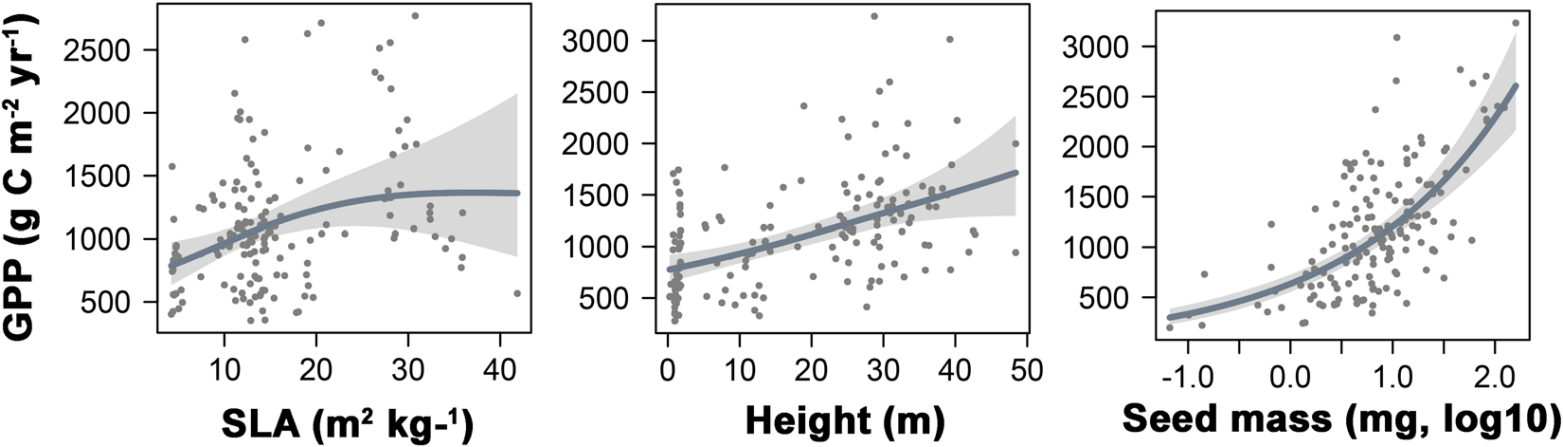
Relationships between annual GPP and the estimated key plant traits. The smoothed functions derived from the fitted generalized additive models (GAMs) show the GPP response to variations in the physical plant traits (summary statistics for the smoothed GAM functions are in Table S5). Shaded areas represent the 95% confidence intervals of the functional relationships. Black dots represent partial residuals.

We use the GAM framework to explain spatial variation in annual GPP measured from 164 globally distributed flux towers as a function of the selected key plant traits. The resulting model explains 66.4% of the variance in annual GPP among tower sites, resulting in model RMSE performance of 403 g C m^−2^ yr^−1^ (Figure S7). Model validation using leave-one-out cross validation indicates RMSE performance of 431 g C m^−2^ yr^−1^. The GPP model RMSE uncertainty is within approximately 32% of the estimated annual carbon flux. The partial correlation function plots show positive relationships between annual GPP and canopy height and seed mass (Fig. 4). Our results also show that seed mass (*r*^2^ = 0.48, *p* < 0.0001) followed by height (*r*^2^ = 0.2, *p < *0.0001), and SLA (*r*^2^ = 0.13, *p* = 0.0003) are the best predictors in explaining the variability in ecosystem productivity. Canopy height differentiates between forest and grassland areas, while seed mass distinguishes between plants in more productive (heavier seeds) to less productive regions (lighter seeds)^26,27^. Annual GPP is generally higher in forests than in grassland (high SLA) biomes even though the photosynthetic capacity of forests may be lower^22^. Likewise, while plants with higher SLA tend to have higher leaf nitrogen content and higher photosynthetic capacity^14^, the total productivity of grasslands with high SLA is generally less than forested regions with generally lower SLA (e.g. boreal forests).

We used the predicted plant trait maps under future (2070) climate conditions to estimate the potential ecosystem productivity response to these changes. Our estimation of current global GPP indicates that terrestrial ecosystems can acquire 134.19 ± 14.3 PgC yr^−1^, while our near future (2070) model projections show a 7.92 ± 1.66% decline in global GPP to 123.56 ± 13.4 PgC yr^−1^. This decline coincides with larger offsetting regional changes in productivity. By 2070, annual GPP above 45 degrees N is expected to increase by 31% (+8.1 ± 0.5 Pg C) from current conditions due to greater dominance of trees and shrubs in northern temperate and boreal zones. However, the productivity increase in northern latitudes will be more than offset by a 17.9% GPP decline in the tropics (−11.8 ± 0.84 PgC yr^−1^) as a result of new environmental conditions that will be suitable for trees with shorter height and lower SLA.

With warmer temperatures in arctic and boreal regions, the length of the growing season is expected to increase, relaxing cold temperature constraints in Arctic ecosystems and promoting higher productivity^8,47^. These changes also favor greater leaf area and canopy height, thus promoting a general increase in woody shrubs and trees, consistent with reported northern greening trends indicated from long-term satellite observation records^48^ and increased tundra shrub abundance^6^. Our results are also consistent with paleo data records showing that during the Pliocene era, when average global temperatures were as high as what is projected for the near future, the high arctic was a suitable habitat for vascular tree species, including larch, spruce, cedar, alder and birch^49^. Greater tree and shrub dominance in the tundra zone may promote increases in above ground carbon storage over the long-term compared to current conditions. Greater tree and shrub dominance may also alter the land surface albedo in ways that promote further temperature and productivity increases^50^.

Warmer temperatures and less precipitation in the tropics indicated from the CMIP5 projections are predicted to lead to shorter trees with lower SLA (Fig. 3) consistent with the strong effect of moisture on plant height and on leaf traits related to water conservation. These results are consistent with reported decreases in SLA of tropical forests as a result of recent environmental change^51^. The projected changes in plant height and SLA favor lower canopy water losses from transpiration, which may have a profound effect on the seasonal water cycle of the Amazon forests as the start of the rainy season is partly due to water transpired by trees^52^. Recent drought events in tropical forests have significantly affected ecosystem productivity^34^ and increased the mortality of trees^53^. Our results show the projected changes in GPP and underlying plant traits for the Amazon tropical forests are significantly larger than other tropical forests in Africa and Southeast Asia. The variable response of these tropical ecosystems is consistent with regional differences in hydroclimatic controls on productivity and associated plant community adaptations to drought^54^.

**Figure 5:**
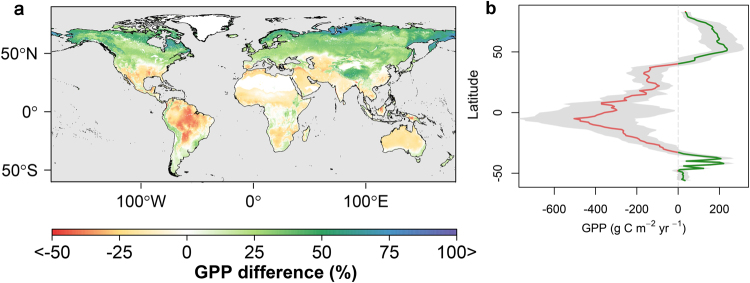
Difference in predicted annual GPP between future and current climate conditions. (**a**) The map shows the projected (year 2070) GPP difference from the current productivity estimates, where GPP is estimated using a generalized additive model and plant traits as explanatory variables, and GPP records from 164 global flux tower sites. (**b)** Mean latitudinal distribution (solid line) of the estimated GPP differences between future (2070) and current conditions; gray shading denotes the standard deviation among GPP models estimated using traits predicted from the 17 different climate model projections. Average GPP decreases at low to mid-latitudes, while higher latitude ecosystems show general productivity increases under projected future climate conditions. Figure 5a was created using the rasterVis library^81^ in R^77^.

Our results indicate that tropical forests will sequester less carbon in the future than they do now due to shifts in plant community structure driven by a warmer and drier climate (Fig. 5). Our GPP estimates for current climate conditions are in the mid-range of other global GPP estimates derived from multiple models (106–175 Pg C yr^−1^)^33–36^. However, our GPP estimate is about 20% larger than the average annual productivity level indicated from a satellite remote sensing data record (MODIS-MOD17)^34^ and 10% higher than the productivity level indicated from a flux tower up-scaled data record (GPP-MTE)^55^ (Figures S7 and S8). We compared our projected future GPP with GPP outputs from five CIMP5 models (Table S8) derived with and without considering CO_2_ fertilization effects (Figure S11). In this study, we did not account for the influence of rising atmospheric CO_2_ levels on plant productivity, and our trait based GPP focus reflects underlying shifts in plant traits in response to climate. The lack of a direct CO2 fertilization effect in our predictions may partially account for the lower trait based annual GPP under future climate conditions (123.56 ± 13.4 Pg C) relative to the average CMIP5 outputs that represent CO_2_ fertilization (155.24 ± 27.5 Pg C). However, our projected future GPP is about 16.7% higher than CIMP5 model GPP estimates that do not consider CO_2_ fertilization. The long-term effect of atmospheric CO_2_ increases on productivity are not well understood, and ongoing studies, including Free Air CO_2_ Enrichment (FACE) experiments, indicate a non-uniform plant response to CO_2_ increases^56,57^. However, the plant trait relationship with local climate^15,58–60^, and the alteration of plant species ranges and structural traits as a result of recent climate change has been observed^61^. In arid lands for example, it has been reported that certain shrubs can reduce their size during dry climate conditions^62^. These trait specifications coincide with changes in productivity in arid regions, and contribute to inter-annual variability of the global carbon cycle^63^.

We found that potential shifts in the geography of plant traits strictly as a result of changing climate conditions and habitat suitability contribute to both enhanced ecosystem productivity at higher latitudes, and reduced productivity over lower latitudes. The net effect of these changes with respect to uncertainty in our trait based GPP estimate is a relatively small reduction in global productivity under projected near-term climate change, which represents a departure from a generally increasing productivity trend since the mid-1970’s^64^.

Plant productivity has been used as a biospheric indicator of ecosystem goods and services^65^. The estimated shift in GPP patterns indicates potential shifts in ecosystem services for much of the global human population. The recent 2015–2016 extensive drought in Somalia that affected about 3.2 million people due to food insecurity and caused an estimated 766,000 displacements^66^ alludes to potential future climate related food insecurity crises at global scales. Based on the current global population distribution^67^, approximately 2.6 billion people are located in areas with projected increases in GPP and could potentially benefit from associated increases in ecosystem goods and services. However, areas with more than five percent projected reduction in GPP are currently inhabited by 4.6 billion people. These areas face potentially higher sustainability risks and security risks exacerbated by both greater ecosystem stress and projected population increases under near future climate.

This study includes several simplifying assumptions that contribute to uncertainty in the model predictions. Potential sources of model uncertainty include our use of a relatively small set of general plant traits to represent changes in plant community characteristics over a global domain. Our approach is also based on the assumption of stable trait-environment relationships for both spatial and future projections, which may not hold. Under climate change, species may go extinct or adapt to fill new environmental spaces beyond their current niche^37^, which may in turn alter relationships between plant traits and environmental conditions. Our future trait projection model approach also neglects intraspecific variability and species turnover^68^ and plant physiological responses and successional processes to changes in climate, which may further affect ecosystem processes including productivity. The use of seed mass (SM) as a driving variable to predict productivity is based on the observed strong empirical correlation between SM and GPP, even though SM is more likely to be a response variable rather than a physical driver of productivity changes. Despite these uncertainties, our results indicate that climate change has the potential to alter plant community structure and the global magnitude and distribution of ecosystem productivity. These changes will influence potential climate feedbacks, plant-animal interactions and ecosystem services. The findings and resulting data products from this research also provide spatially explicit plant trait information that may help to better inform the representation of plant traits in global ecosystem models that extend beyond general assumptions of biome level homogeneity.

## Methods

We used 19 climatic variables from WorldClim database^41^ to explain spatial variability in three major physical plant traits informed by the global plant traits database (TRY)^40^. Key plant traits from TRY used in our study included SLA (m^2^ kg^−1^), tree height (m) and seed mass (mg). The selected WorldClim climatic variables are derived from global average long term (1950–2000) monthly precipitation and mean daily minimum and maximum air temperatures from global weather station records^41^. The climatic variables analyzed included: annual mean temperature and mean diurnal range, Isothermality, temperature seasonality, maximum temperature of the warmest month, minimum temperature of the coldest month, temperature annual range, mean temperature of wettest quarter, mean temperature of driest quarter, mean temperature of warmest quarter, mean temperature of coldest quarter, annual precipitation, precipitation of wettest month, precipitation of driest month, precipitation seasonality, precipitation of wettest quarter, precipitation of driest quarter, precipitation of warmest quarter and precipitation of coldest quarter.

The WorldClim variables are mapped to a global grid at 30 arc-second resolution. These data are spatially interpolated from 47,554 and 24,542 global weather stations for precipitation and temperature, respectively, and have been used extensively for analyzing species habitat relationships and ecological studies (e.g.^69–73^). In this dataset, current climate is characterized by the average of monthly climatic variables from 1950–2000.

We used 204,504 global observations of key plant traits (Figure S1) representing dominant vegetation type characteristics from the TRY database^40^. TRY database covers a high fraction of the most frequent or dominant species available in sPlot^74^, the largest repository for plant community data in the world. To ensure that the trait observations represent global biomes, we used the site level documentation provided in the global plant traits database including woodiness and growth form information to select dominant species traits representing site level plant functional type (PFT) categories that matched collocated general PFT classes represented within a global land cover classification (MODIS MOD12 land cover product^75^). This global selection process resulted in 10,327 SLA observations from 2,343 dominant species, 5,417 plant height observations from 2,188 species, and 2000 seed mass observations from 1,275 species (Table S1, Figure S2) including 6 observations for crops. In the case of multiple observations per location, we used weighted median values of observations based on their functional types, which resulted in 952 observations of seed mass, 1,042 observations of SLA and 1,028 observations of canopy height.

We used a Generalized Additive Model (GAM) framework^43^ to describe spatial patterns of plant traits within global biomes, and validated the models using a leave-one-out cross validation method. We assumed that temperature and precipitation are effective proxies for respective energy and water constraints to vegetation processes, and can explain global variability in functional plant traits. We used the visreg library^76^ in the R programing environment^77^ to show the relationship between response and explanatory variables in our global GAM. This process revealed the relationship between each explanatory and response variable while other covariates were held fixed. The difference between a generalized linear model (GLM) and the GAM approach is that the GAM adds smoothed non-parametric functions to the parametric part of a GLM^43^, allowing for greater flexibility and improved fit^78^ in the model structure:

1 $$g({{\rm{\mu }}}_{i})={X}_{i}^{\ast }\theta \,+{f}_{1}({X}_{1i})+{f}_{2}({X}_{2i})+{f}_{3}({X}_{3i})+\ldots $$

where ${{\rm{\mu }}}_{i}$ ≡ E (*Y*_i_) and the response variable *Y*_i_ follows an exponential family distribution; X_*i*_ is ^th^e *i*^th^ row of the model matrix, and *θ* is a corresponding parameter vector; *f*_*i*_ are smoothed functions of the covariates in X_*i*_. Because the PFTs are distinguishable using physical plant traits^23^, we used PFT as a dummy variable in the GAM. In order to minimize co-linearity effects in the regression models, among predictor variables with more than 70% correlation, only one variable was retained, and the rest were excluded from the models. In addition to climatic variables, we used soil attributes including soil organic carbon, clay and silt content, and soil pH to test their predictive power in explaining the variance in trait data, and tested the traits models with and without using the soil attribute data. We optimized the models using stepwise variable selection by means of the AIC to choose the best explanatory variable for prediction of the selected plant traits (Table S4). In order to reduce overfitting of the regression models, we reduced the number of nods in the smoothed functions and used a restricted maximum likelihood estimator.

Future climate projections are available for climatic variables downscaled from global ESM climate simulations from the recent IPCC CMIP5^79^ assessment. We used future ESM climate projections from the RCP 8.5 of the A2 emission scenario^42^ (Table S2), where the future climate conditions represent model averages for the 2061–2080 time period centered on year 2070. We used fitted models of the plant traits spanning all vegetated land areas to create global maps of the selected plant traits under current climate (Figure S3), and projected future climate conditions based on each of 17 CMIP5 climate models and their ensemble mean (Figure S5).

We used daily GPP measurements from 164 flux towers from the global FLUXNET network (Table S5) to calculate the annual GPP climatology (g C m^−2^ yr^−1^) for sites representing major global biomes (Figure S4). We explained spatial variability in annual GPP from multi-year observations across the tower sites, using the extrapolated trait maps for current and future climate conditions as explanatory variables (Table S6, Figure S7), and predicted the global annual GPP based on the plant trait distributions (Figure S8) using the GAM. Areas having less than 50 mm of annual precipitation and representing deserts and other barren land were eliminated from the analysis. We also compared the GPP estimates derived from the predicted plant traits information with alternative GAM based GPP predictions derived using only the climate variables (Table S7). Additionally, we compared our GAM predicted annual GPP results with two other global productivity datasets for model verification, including the average annual MODIS MOD17A3 (Collection 5) GPP data record derived at 1-km spatial resolution for the 2000–2014 period^34^ and a global tower observation up-scaled GPP record derived at 0.5 degree spatial resolution from 2000–2011 (MTE GPP)^55^. We also calculated the annual GPP spatial means for each 0.05-degree latitudinal bin from these global datasets, while the GPP estimates for future climate conditions were compared against alternative global GPP projections obtained from five CMIP5 global ESMs (Table S8) derived with and without considering CO_2_ fertilization effects.

We acquired global human population data from the NASA Socioeconomic Data and Applications Center (SEDAC)^80^. The SEDAC data provide human population estimates for the year 2000 in each grid cell over the global domain with a 2.5 arc-minute (~0.04 degree) spatial resolution. We classified our global GPP estimates into two regions representing grid cells with at least 5% increase and more than 5% decrease in productivity under projected future (2070) conditions. We then determined the human population densities within each region.

### Data availability

All data used in this research are publicly available from the cited literature. Results and data products generated from this research are publicly available for download from NTSG and the University of Montana or through contact with the corresponding author.

## Electronic supplementary material

Supplementary Information
