# Supplementary material for: Future global productivity will be affected by plant trait response to climate
Source: Sci Rep. 2018 Feb 12;8:2870. doi: 10.1038/s41598-018-21172-9 (PMC5809371; doi:10.1038/s41598-018-21172-9)
Supplement: Supplementary file 1 — Supplementary Information [file 41598_2018_21172_MOESM1_ESM.docx]

## Supporting Information

**Future global productivity will be affected by plant trait response to climate**

Authors: Nima Madani, John S. Kimball, Ashley P. Ballantyne, David L.R. Affleck, Peter M. van Bodegom, Peter B. Reich, Jens Kattge, Anna Sala, Mona Nazeri, Matthew O. Jones, Maosheng Zhao, Steven W. Running

**Supplementary Tables:**

**Table S1.** Number of individual observations selected from the global TRY database for each physical plant trait ^1–46^ used for developing the GAM prediction framework in this study.

| Category | Sub category | SLA | SM | Height |
| --- | --- | --- | --- | --- |
| Type | Broadleaf | 1958 | 1106 | 1927 |
|  | Needle leaf | 12 | 4 | 8 |
|  | Deciduous | 367 | 159 | 247 |
|  | Crops | 6 | 6 | 6 |
| Woodiness | Woody | 1690 | 926 | 1832 |
|  | Non-woody | 653 | 349 | 356 |
| Growth form | Tree | 1118 | 546 | 1352 |
|  | Non-tree | 1225 | 729 | 836 |

**Table S2.** CMIP5 global climate models (17) used to derive the ensemble mean of climatic variable projections for the RCP8.5 greenhouse gas scenario for year 2070. The climatic variables for the future climate change scenarios are available from the WorldClim database.

| Model Name | Description | Reference |
| --- | --- | --- |
| ACCESS1-0 | The Australian Community Climate and Earth System Simulator | ^47^ |
| BCC-CSM1-1 | Beijing Climate Center, Climate System Model | ^48^ |
| CCSM4 | Community Climate System Model | ^49^ |
| CNRM-CM5 | Centre National de Recherches Me´te´orologiques | ^50^ |
| GFDL-CM3 | Geophysical Fluid Dynamics Laboratory- coupled general circulation model | ^51^ |
| GISS-E2-R | Goddard Institute for Space Studies | ^52^ |
| HadGEM2-AO | Hadley Centre Global Environment Model version 2. Ocean & Sea-ice | ^53^ |
| HadGEM2-CC | Hadley Centre Global Environmental Model, version 2 (Carbon Cycle) | ^53^ |
| HadGEM2-ES | Hadley Centre Global Environmental Model,  version 2 (Earth System) | ^53^ |
| INMCM4 | Institute of Numerical Mathematics Coupled  Model, version 4.0 | ^54^ |
| IPSL-CM5A-LR | L’Institut Pierre-Simon Laplace Coupled Model,  version 5A, coupled  with NEMO, low resolution | ^55^ |
| MIROC-ESM-CHEM | Model for Interdisciplinary Research on Climate,  Earth System Model, Chemistry Coupled | ^56^ |
| MIROC-ESM | Model for Interdisciplinary Research on Climate,  Earth System Model | ^56^ |
| MIROC5 | Model for Interdisciplinary Research on Climate | ^57^ |
| MPI-ESM-LR | Max Planck Institute Earth System Model,  low resolution | ^58^ |
| MRI-CGCM3 | Meteorological Research Institute- Coupled Global Climate Model | ^59^ |
| NorESM1-M | Norwegian Earth System Model, version 1 | ^60^ |

**Table S3.** Summary statistics for the smoothed function from each final GAM model used to predict the physical plant trait distributions in this study, including degree of freedom, *F* and *P* statistics and adjusted *R^2^*.

| Model | variable | *Est. df* | *Ref.df* | *F* | *P* | *Adj. R^2^* | *RMSE.test* |
| --- | --- | --- | --- | --- | --- | --- | --- |
| SLA | Annual Precip | 4.091 | 9 | 5.534 | < 0.0001 | 0.682 | 3.22 |
|  | Max Temp warmest month | 4.586 | 9 | 2.55 | < 0.0001 |  |  |
|  | Min Temp of coldest month | 2.688 | 9 | 3.897 | < 0.0001 |  |  |
| Height | Annual Precip | 1.916 | 9 | 0.783 | < 0.0001 | 0.662 | 10.07 |
|  | Max Temp warmest month | 6.353 | 9 | 8.592 | < 0.01 |  |  |
|  | Min Temp of coldest month | 4.055 | 9 | 3.545 | < 0.0001 |  |  |
| Seed Mass | Annual Precip | 4.567 | 9 | 13.649 | < 0.0001 | 0.455 | 0.8 |
|  | Mean temp of warmest quarter | 2.881 | 9 | 5.672 | < 0.0001 |  |  |

**Table S4.** The Akaike Information Criterion (AIC) estimated for each trait model with and without using soil attribute data^61^ including soil silt content, clay content, organic carbon and PH at 1 m depth.

| Trait | Model | AIC |
| --- | --- | --- |
| SLA | Climate + Soil  Climate | 6448  6461 |
| Height | Climate + Soil  Climate | 3403  3388 |
| Seed Mass | Climate +Soil  Climate | 789  781 |

**Table S5.** List of all flux tower sites used for predicting global gross primary productivity; key references from the literature for many of the tower sites are also shown.

| Name | LOCATION_LONG | LOCATION_LAT | IGBP | Reference |
| --- | --- | --- | --- | --- |
| AR-Vir | -56.1886 | -28.2395 | ENF |  |
| AT-Neu | 11.3175 | 47.1167 | GRA |  |
| AU-Ade | 131.1178 | -13.0769 | WSA |  |
| AU-ASM | 133.249 | -22.283 | ENF |  |
| AU-Cpr | 140.5891 | -34.0021 | SAV |  |
| AU-Cum | 150.7225 | -33.6133 | EBF |  |
| AU-DaP | 131.3181 | -14.0633 | GRA |  |
| AU-DaS | 131.3881 | -14.1593 | SAV |  |
| AU-Dry | 132.3706 | -15.2588 | SAV |  |
| AU-Emr | 148.4746 | -23.8587 | GRA |  |
| AU-Fog | 131.3072 | -12.5452 | WET |  |
| AU-GWW | 120.6541 | -30.1913 | SAV |  |
| AU-RDF | 132.4776 | -14.5636 | WSA |  |
| AU-Rig | 145.5759 | -36.6499 | GRA |  |
| AU-Rob | 145.6301 | -17.1175 | EBF |  |
| AU-Tum | 148.1517 | -35.6566 | EBF | ^62^ |
| AU-Whr | 145.0294 | -36.6732 | EBF |  |
| BE-Bra | 4.5206 | 51.3092 | MF | ^62^ |
| BE-Jal | 6.07333 | 50.5639 | MF | ^62^ |
| BE-Lon | 4.7461 | 50.5516 | CRO | ^62^ |
| BE-Vie | 5.9981 | 50.3051 | MF | ^63^ |
| BR-Ban | -50.1591 | -9.82442 | EBF | ^64^ |
| BR-Cax | -51.459 | -1.71972 | EBF | ^65^ |
| BR-Ma2 | -60.2093 | -2.6091 | EBF | ^64^ |
| BR-Sa1 | -54.9589 | -2.85667 | EBF | ^66^ |
| BR-Sa3 | -54.9714 | -3.01803 | EBF | ^62^ |
| CA-Gro | -82.1556 | 48.2167 | MF | ^62^ |
| CA-Let | -112.94 | 49.7093 | ENF | ^64^ |
| CA-NS1 | -98.4839 | 55.8792 | ENF | ^62^ |
| CA-Obs | -105.118 | 53.9872 | ENF | ^67^ |
| CA-Qcu | -74.0365 | 49.2671 | ENF | ^64^ |
| CA-Qfo | -74.3421 | 49.6925 | ENF | ^64^ |
| CA-SF1 | -105.818 | 54.485 | ENF | ^67^ |
| CA-SF2 | -105.878 | 54.2539 | ENF |  |
| CA-SF3 | -106.005 | 54.0916 | OSH |  |
| CA-WP1 | -112.467 | 54.9538 | ENF | ^68^ |
| CH-Cha | 8.4104 | 47.2102 | GRA |  |
| CH-Fru | 8.5378 | 47.1158 | GRA | ^69^ |
| CH-Oe1 | 7.7319 | 47.2858 | GRA | ^64^ |
| CN-Cha | 128.0958 | 42.4025 | MF |  |
| CN-Cng | 123.5092 | 44.5934 | GRA |  |
| CN-Dan | 91.0664 | 30.4978 | GRA |  |
| CN-Din | 112.5361 | 23.1733 | EBF |  |
| CN-Du2 | 116.2836 | 42.0467 | GRA | ^62^ |
| CN-Ha2 | 101.3269 | 37.6086 | WET |  |
| CN-HaM | 101.2988 | 37.59583 | GRA | ^64^ |
| CN-Qia | 115.0581 | 26.7414 | ENF |  |
| CN-Xfs | 116.2797 | 43.55444 | GRA |  |
| CN-Xi1 | 116.6778 | 43.54583 | GRA |  |
| CZ-BK1 | 18.5384 | 49.5026 | ENF | ^70^ |
| CZ-BK2 | 18.5447 | 49.4953 | MF | ^71^ |
| DE-Akm | 13.6834 | 53.8662 | WET |  |
| DE-Geb | 10.9143 | 51.1001 | CRO | ^64^ |
| DE-Gri | 13.5125 | 50.9495 | GRA | ^62^ |
| DE-Hai | 10.453 | 51.0792 | DBF | ^62^ |
| DE-Kli | 13.5225 | 50.8929 | CRO | ^62^ |
| DE-Lkb | 13.3047 | 49.0996 | ENF | ^62^ |
| DE-Meh | 10.6555 | 51.2753 | MF | ^62^ |
| DE-Obe | 13.7196 | 50.7836 | ENF |  |
| DE-RuS | 6.4472 | 50.8659 | CRO |  |
| DE-Spw | 14.0337 | 51.8923 | WET |  |
| DE-Tha | 13.5669 | 50.9636 | ENF | ^62^ |
| DK-Fou | 9.58722 | 56.4842 | CRO | ^62^ |
| DK-NuF | -51.3861 | 64.1308 | WET |  |
| DK-Ris | 12.0972 | 55.5303 | CRO | ^72^ |
| DK-Sor | 11.6446 | 55.4859 | DBF |  |
| ES-LgS | -2.9658 | 37.0979 | OSH |  |
| ES-VDA | 1.4485 | 42.1522 | GRA | ^73^ |
| FI-Hyy | 24.295 | 61.8475 | ENF |  |
| FI-Jok | 23.5135 | 60.8986 | CRO |  |
| FI-Kaa | 27.295 | 69.1407 | GRA | ^62^ |
| FI-Sod | 26.6378 | 67.3619 | ENF | ^62^ |
| FR-Aur | 1.107778 | 43.54944 | CRO | ^62^ |
| FR-Fon | 2.780147 | 48.47634 | DBF | ^62^ |
| FR-Gri | 1.9519 | 48.8442 | CRO | ^62^ |
| FR-Hes | 7.06462 | 48.67083 | DBF | ^62^ |
| FR-LBr | -0.76819 | 44.72083 | ENF | ^62^ |
| FR-Lq1 | 2.73703 | 45.6441 | GRA |  |
| FR-Pue | 3.592775 | 43.7375 | EBF | ^62^ |
| GF-Guy | -52.9119 | 5.279167 | EBF |  |
| HU-Mat | 19.72426 | 47.84583 | CRO |  |
| ID-Pag | 114.036 | 2.345 | EBF | ^62^ |
| IE-Dri | -8.75181 | 51.9867 | GRA |  |
| IT-Amp | 13.6052 | 41.9041 | GRA |  |
| IT-Bon | 16.5347 | 39.4778 | ENF |  |
| IT-CA2 | 12.026 | 42.3772 | CRO |  |
| IT-Col | 13.5881 | 41.8494 | DBF | ^62^ |
| IT-Cp2 | 12.3573 | 41.7043 | EBF |  |
| IT-Cpz | 12.3761 | 41.7052 | EBF | ^62^ |
| IT-Isp | 8.6336 | 45.8126 | DBF |  |
| IT-Mal | 11.7028 | 46.1167 | GRA |  |
| IT-Noe | 8.1515 | 40.6061 | CSH |  |
| IT-PT1 | 9.06104 | 45.2009 | CRO | ^62^ |
| IT-Ren | 11.4337 | 46.5869 | ENF |  |
| IT-Ro2 | 11.9209 | 42.3903 | DBF | ^62^ |
| IT-Tor | 7.5781 | 45.8444 | GRA |  |
| JP-MBF | 142.3186 | 44.3869 | DBF |  |
| JP-SMF | 137.0788 | 35.2617 | MF |  |
| JP-Tak | 137.423 | 36.1462 | DBF |  |
| JP-Tef | 142.1062 | 45.05634 | MF |  |
| JP-Tom | 141.5149 | 42.7395 | MF |  |
| KR-Hnm | 126.57 | 34.55 | CRO |  |
| MY-PSO | 102.3062 | 2.973 | EBF |  |
| NL-Ca1 | 4.927 | 51.971 | GRA |  |
| NL-Hor | 5.0713 | 52.2404 | GRA |  |
| NL-Loo | 5.7436 | 52.1666 | ENF | ^62^ |
| NL-Mol | 4.639 | 51.65 | CRO |  |
| PT-Mi2 | -8.02455 | 38.4765 | GRA | ^62^ |
| RU-Che | 161.339 | 68.6147 | OSH | ^62^ |
| RU-Cok | 147.4943 | 70.8291 | OSH | ^62^ |
| RU-Fyo | 32.9221 | 56.4615 | ENF |  |
| RU-Ha1 | 90.0022 | 54.7253 | GRA | ^72^ |
| RU-Zot | 89.35597 | 60.80417 | ENF |  |
| SD-Dem | 30.4783 | 13.2829 | SAV |  |
| SE-Deg | 19.56889 | 64.1875 | ENF |  |
| SE-Fla | 19.45883 | 64.1125 | ENF | ^62^ |
| SE-Sk1 | 17.92385 | 60.12083 | ENF | ^62^ |
| SE-Sk2 | 17.83569 | 60.1125 | ENF | ^62^ |
| UK-Ham | -0.85633 | 51.12083 | DBF | ^62^ |
| US-AR1 | -99.42 | 36.4267 | GRA |  |
| US-AR2 | -99.5975 | 36.6358 | GRA |  |
| US-ARM | -97.4888 | 36.6058 | CRO |  |
| US-Atq | -157.409 | 70.4696 | GRA |  |
| US-Bar | -71.2881 | 44.0646 | DBF | ^62^ |
| US-Bkg | -96.8362 | 44.3453 | GRA | ^62^ |
| US-Blo | -120.633 | 38.8952 | ENF |  |
| US-Bn1 | -145.378 | 63.9198 | ENF |  |
| US-Bo1 | -88.2904 | 40.0062 | CRO |  |
| US-Dk3 | -79.0942 | 35.9782 | MF | ^62^ |
| US-Fmf | -111.727 | 35.1426 | ENF |  |
| US-FR2 | -97.9962 | 29.9495 | WSA | ^62^ |
| US-Fuf | -111.762 | 35.089 | ENF | ^62^ |
| US-Goo | -89.8735 | 34.2547 | CRO | ^74^ |
| US-Ha1 | -72.1715 | 42.5378 | DBF |  |
| US-Ho1 | -68.7402 | 45.2041 | MF |  |
| US-Ho2 | -68.747 | 45.2091 | ENF | ^62^ |
| US-Ivo | -155.768 | 68.4875 | OSH | ^75^ |
| US-KS2 | -80.6727 | 28.6125 | CSH | ^75^ |
| US-Lin | -119.842 | 36.3566 | CRO |  |
| US-LPH | -72.1934 | 42.54583 | DBF |  |
| US-Me1 | -121.505 | 44.57917 | WSA | ^75^ |
| US-Me2 | -121.557 | 44.4523 | ENF | ^74^ |
| US-Me3 | -121.608 | 44.3154 | ENF | ^62^ |
| US-Me4 | -121.622 | 44.4992 | ENF | ^75^ |
| US-Me6 | -121.608 | 44.3233 | ENF |  |
| US-MMS | -86.4131 | 39.3232 | DBF |  |
| US-Ne1 | -96.4766 | 41.1651 | CRO | ^72^ |
| US-Ne2 | -96.4701 | 41.1649 | CRO | ^62^ |
| US-NR1 | -105.546 | 40.0329 | ENF | ^75^ |
| US-SO2 | -116.623 | 33.3739 | CSH |  |
| US-SO3 | -116.623 | 33.3772 | WSA | ^75^ |
| US-SP3 | -82.1611 | 29.75417 | EBF | ^75^ |
| US-SRM | -110.866 | 31.8214 | WSA | ^76^ |
| US-Syv | -89.3477 | 46.242 | MF |  |
| US-Ton | -120.966 | 38.4316 | WSA | ^77^ |
| US-Tw3 | -121.647 | 38.1159 | CRO |  |
| US-UMB | -84.7138 | 45.5598 | DBF | ^74^ |
| US-Var | -120.951 | 38.4133 | GRA | ^74^ |
| US-WCr | -90.0799 | 45.8059 | DBF | ^74^ |
| US-Wi4 | -91.1663 | 46.7393 | MF | ^62^ |
| US-Wkg | -109.942 | 31.7365 | GRA |  |
| US-Wrc | -121.952 | 45.8205 | ENF | ^74^ |
| ZA-Kru | 31.4969 | -25.0197 | SAV | ^78^ |
| ZM-Mon | 23.2528 | -15.4378 | DBF | ^79^ |

**Table S6.** Summary statistics for the GAM smoothed function used for modeling global GPP using plant trait information.

| Model | variable | *Est. df* | *Ref.df* | *F* | *AIC* | *P* | *Adj. R2* | *RMSE.test* |
| --- | --- | --- | --- | --- | --- | --- | --- | --- |
| GPP | SLA | 1.001 | 1.002 | 77.27 | 2603 | 0.0003 | 0.664 | 431 |
|  | Height | 5.53 | 6.366 | 8.234 | 2595 | < 0.0001 |  |  |
|  | Seed mass | 2.759 | 3.476 | 15.573 | 2513 | < 0.0001 |  |  |

**Table S7.** Summary statistics for models predicting tower GPP using traits versus climate variables.

| Model | R^2^ | RMSE | AIC |
| --- | --- | --- | --- |
| GPP-Traits | 0.66 | 403 | 2454 |
| GPP-Climate | 0.61 | 437 | 2480 |

**Table S8.** List of CMIP5 GPP data used for comparison with future trait based GPP estimates from this study.

| Model Name | Experiment ID | CO_2_ forcing | Vegetation type | Ensemble ID |
| --- | --- | --- | --- | --- |
| GFDL-ESM2M | RCP8.5 | CO_2_/No CO_2_ | Static | r1i1p1 |
| HADGEM2-ES | RCP8.5 | CO_2_/No CO_2_ | Static | r1i1p1 |
| ISPL_CM5A-LR | RCP8.5 | CO_2_/No CO_2_ | Static | r1i1p1 |
| MIROC-ESM-CHEM | RCP8.5 | CO_2_/No CO_2_ | Static | r1i1p1 |
| NORESM1-M | RCP8.5 | CO_2_/No CO_2_ | Static | r1i1p1 |

**Supplementary figures:**


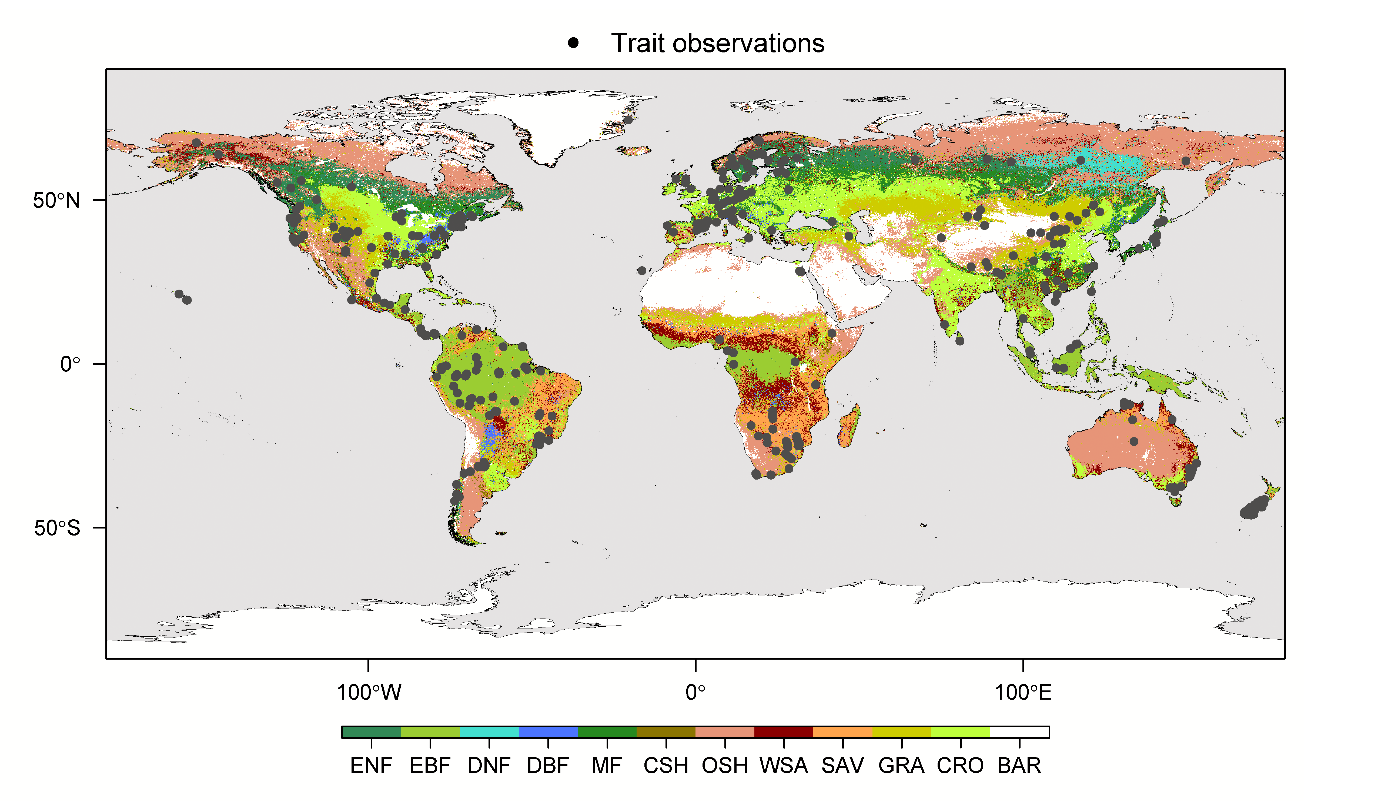


**Figure S1. Global distribution of plant trait observation locations used for the GAM extrapolations.** Plant trait observation sites overlaid on a global land cover map (MODIS MCD12C1-Type2) ^80^ depicting the following PFT classes: Evergreen Needle leaf Forest (ENF); Evergreen Broadleaf Forest (EBF); Deciduous Needle leaf Forests (DNF); Deciduous Broadleaf Forests (DBF); Closed Shrubland (CSH); Open Shrubland (OSH); Woody Savanna (WSA);

Savanna (SAV); Grassland (GRA); Cropland (CRO); Barren or sparse vegetation (BAR). The figure was created in the ArcMap software environment ^81^.


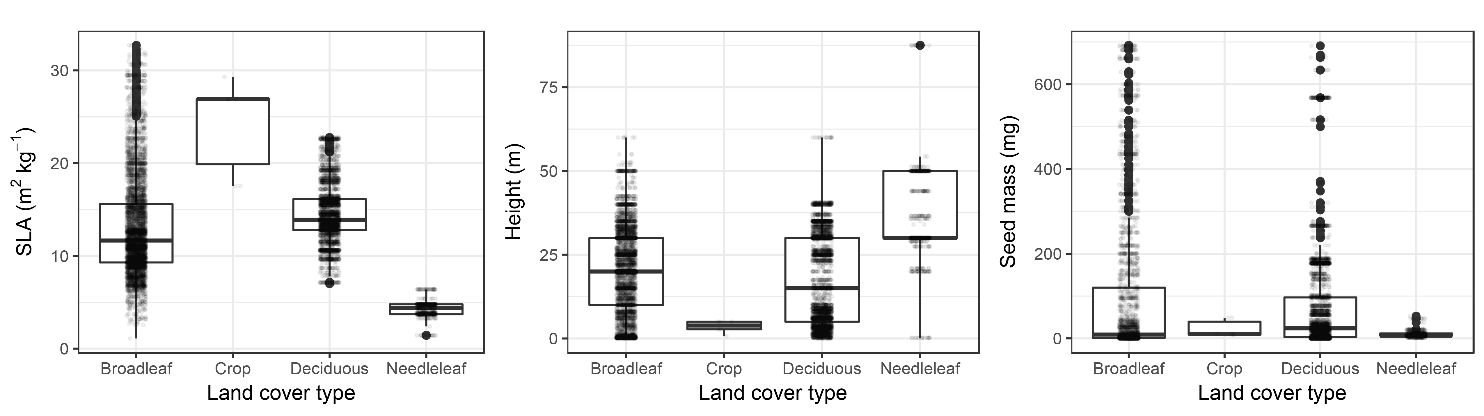


**Figure S2. Global plant trait variations within and among the major land cover classes.** Boxplots showing the variation in key plant traits relative to land cover types reported by site investigators. Black circles on boxplots represent each reported observation.


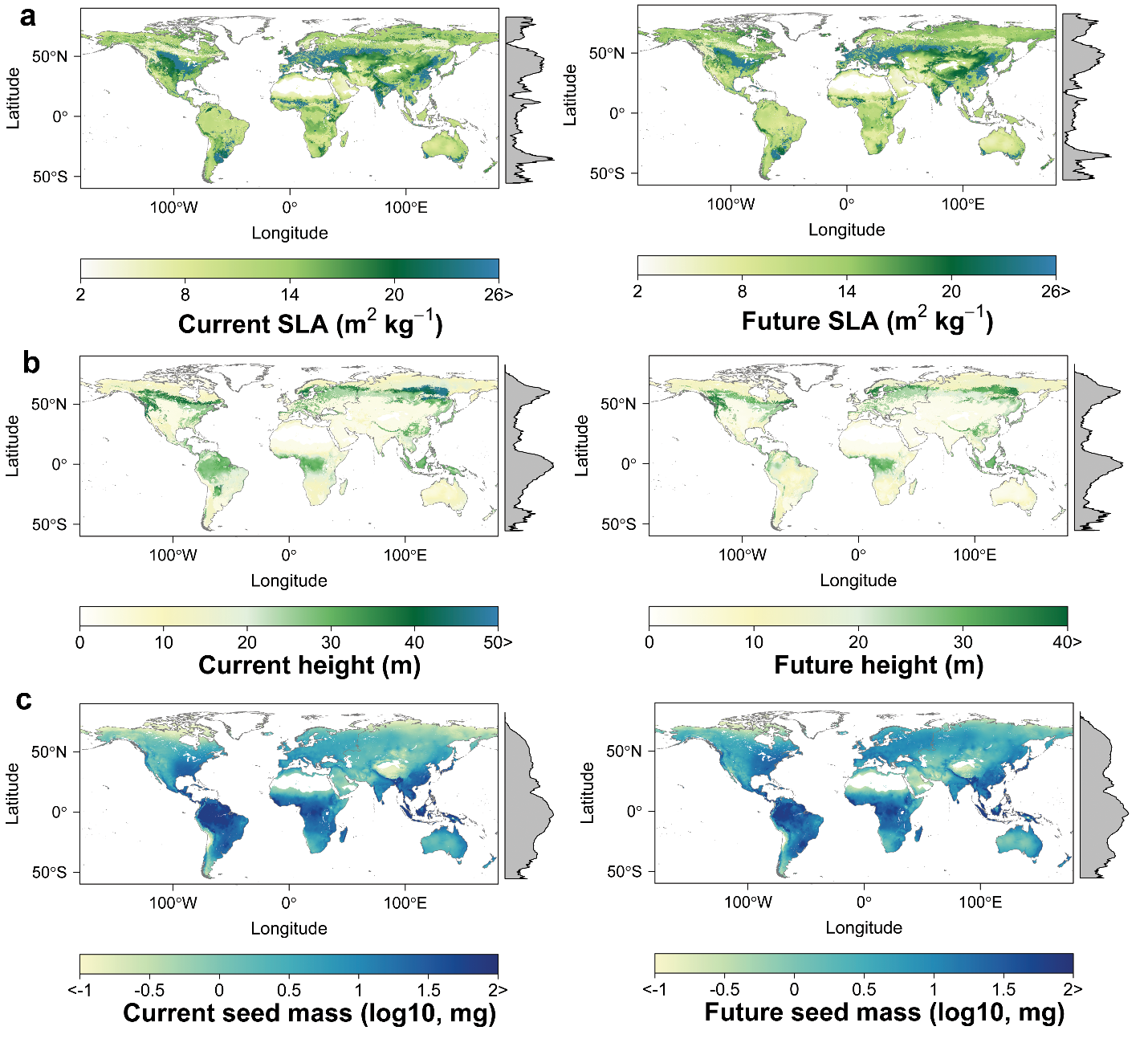


**Figure S3.** Estimated global distributions of key plant traits for SLA (a), canopy height (b), and seed mass (c) based on current (1950-2000) and projected future (2070) climate conditions (ensemble mean). The figure was created using the rasterVis library ^82^ in R ^83^.


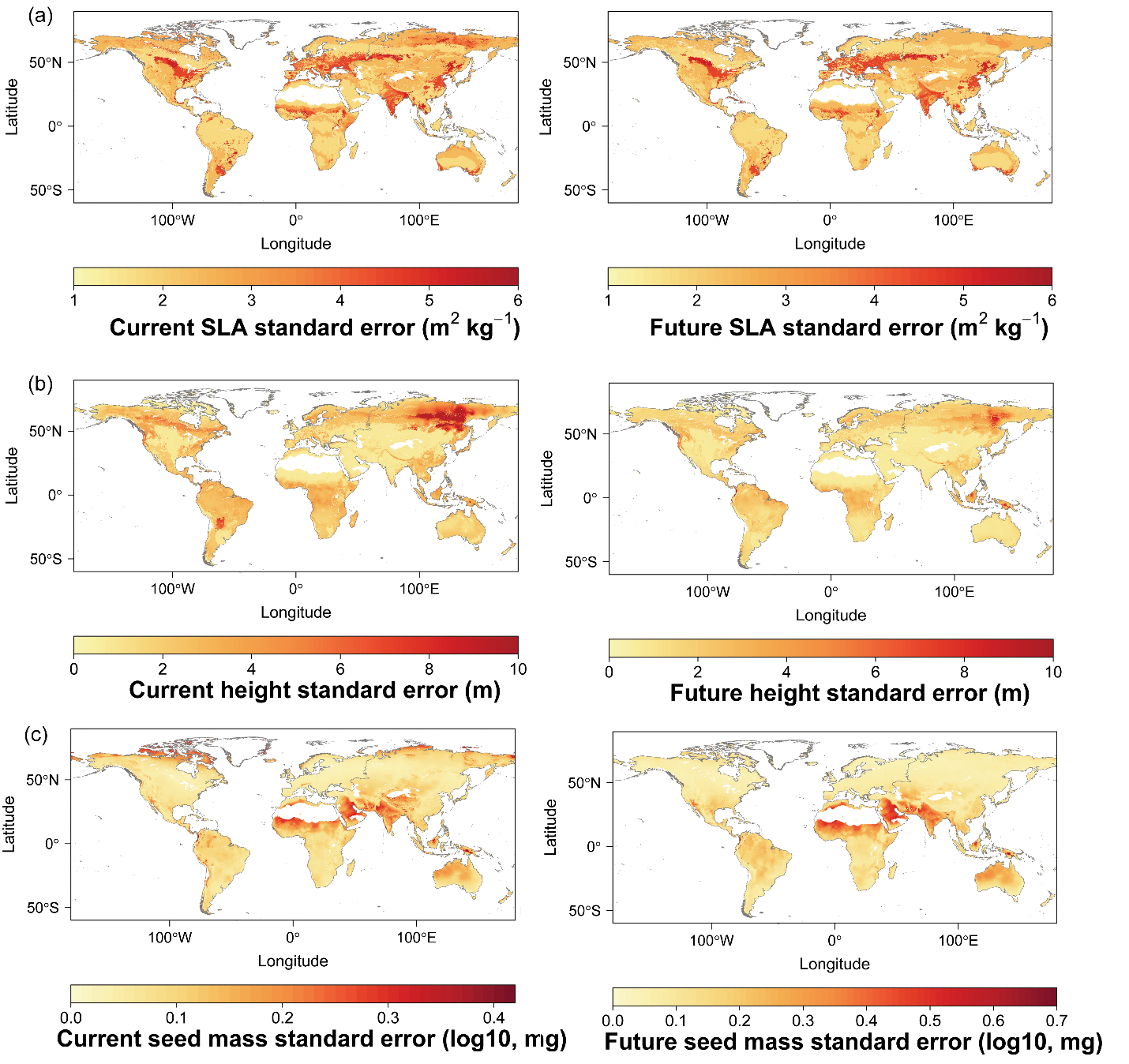


**Figure S4.** The standard error associated with derived traits for current and future climate conditions (ensemble mean) for SLA (a), canopy height (b), and seed mass (c). The figure was created using the rasterVis library ^82^ in R ^83^.


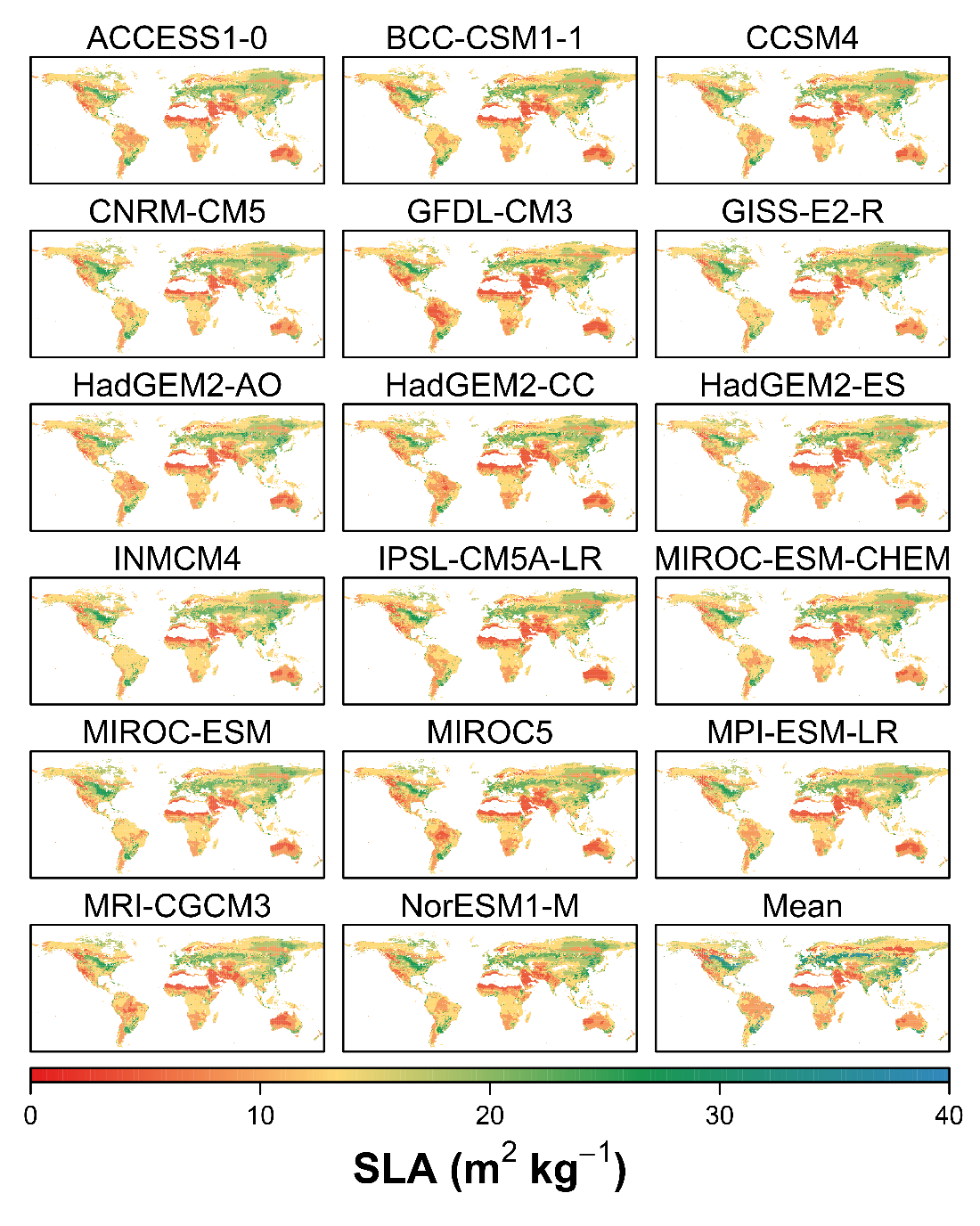


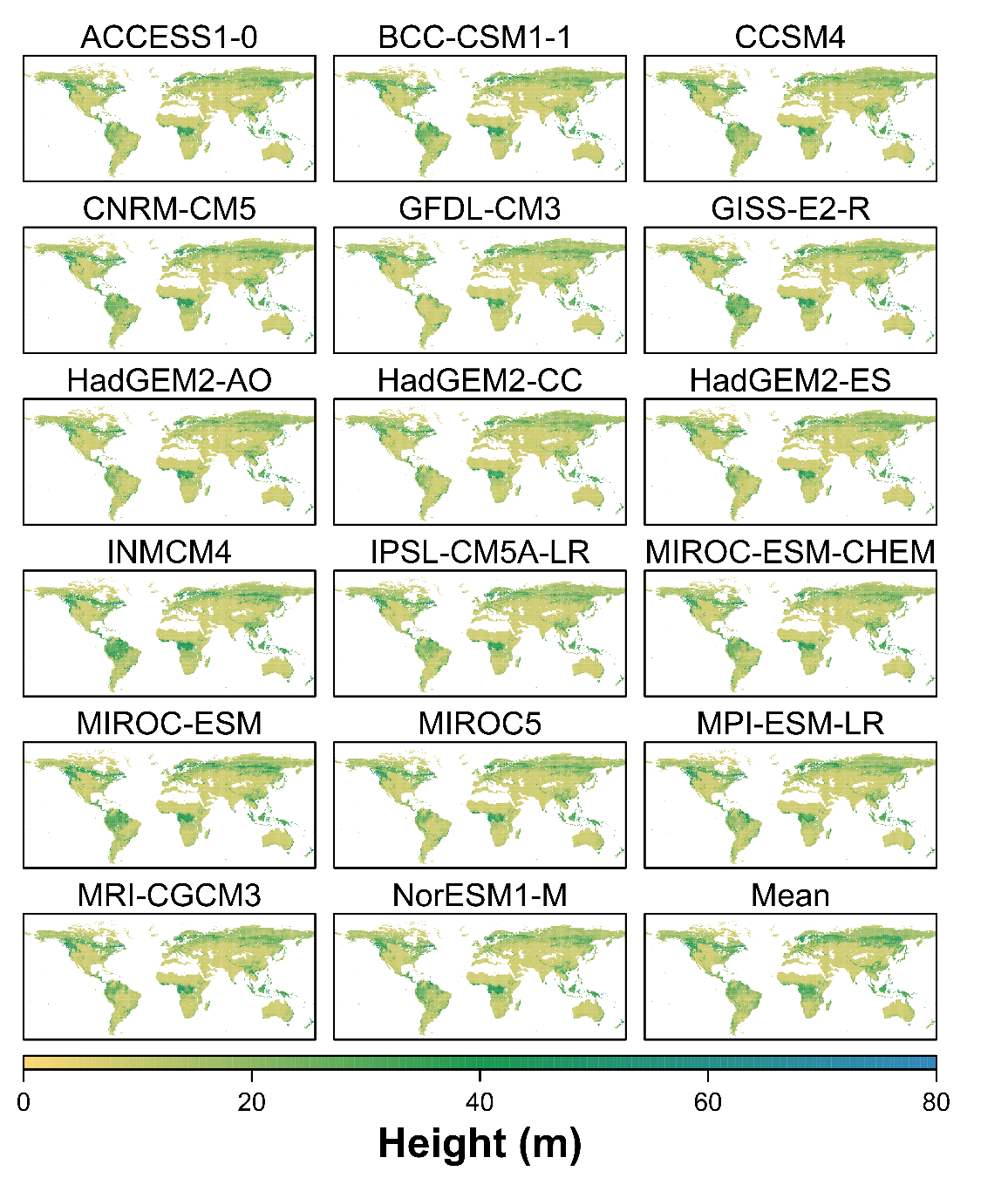


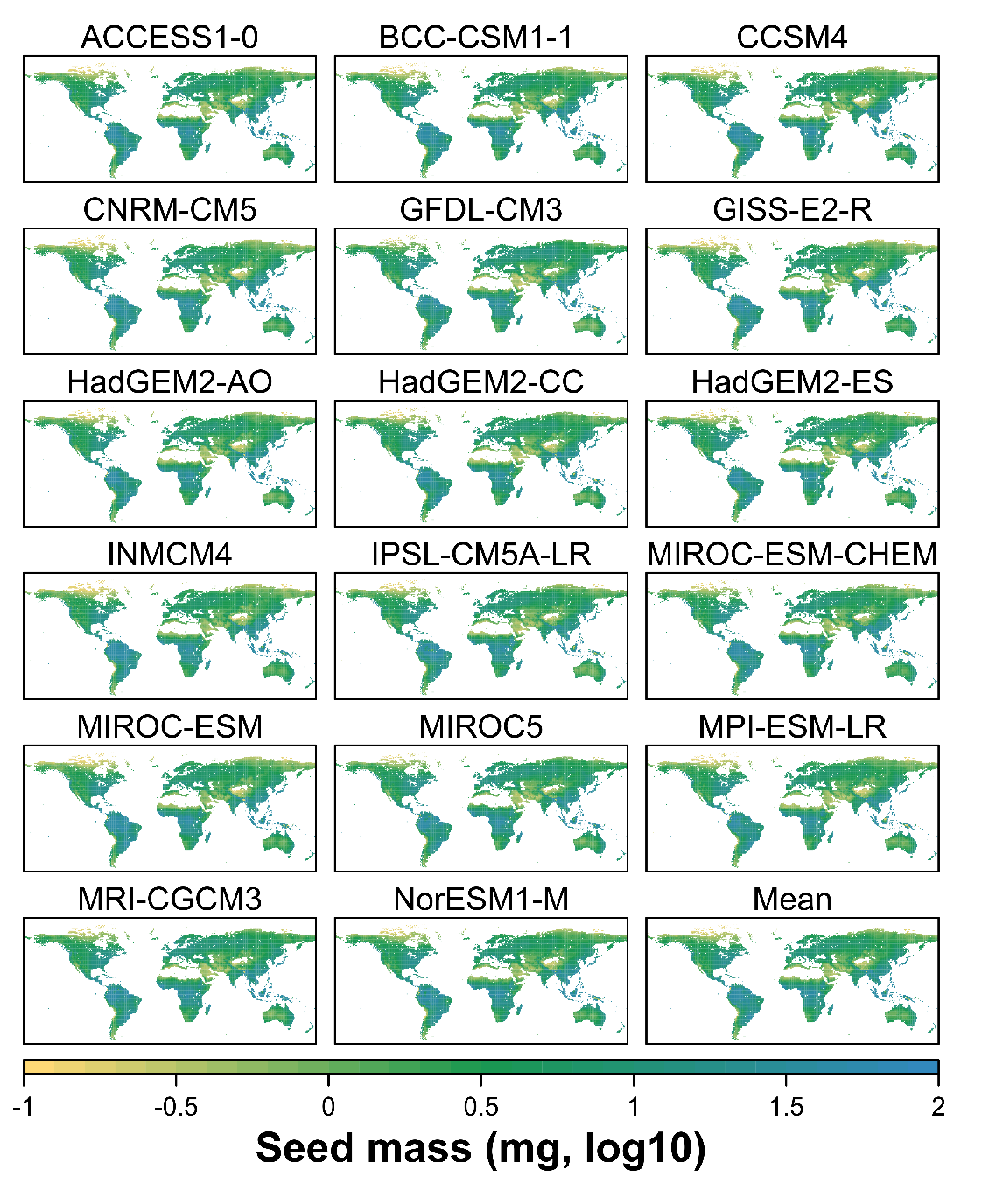


**Figure S5.** Projected changes in key plant key traits based on individual CMIP5 climate model projections and their ensemble mean. The mean climate-trait relationship is used to model predicted changes in community plant traits in the near future. The figure was created using the rasterVis library ^82^ in R ^83^.


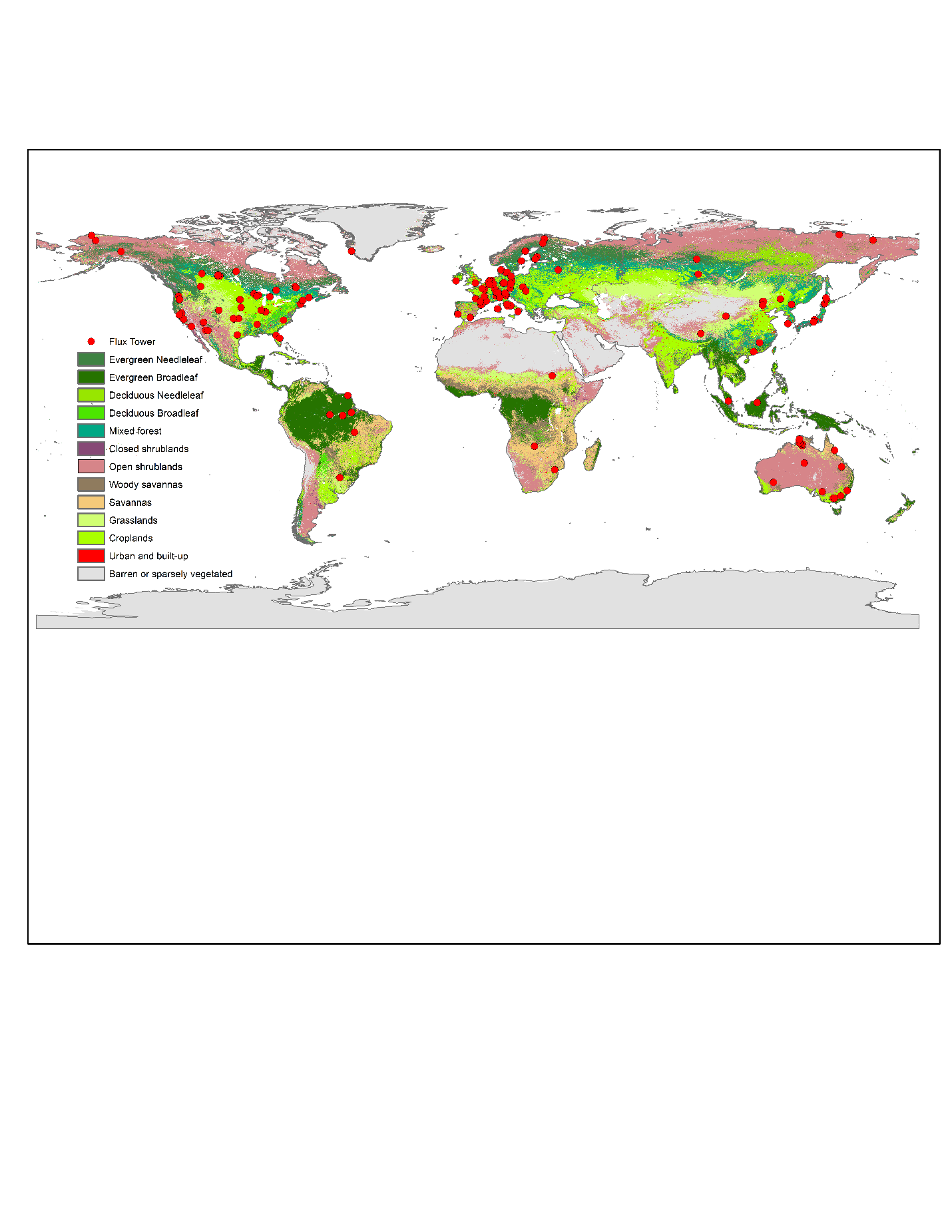


**Figure S6.** Locations of 164 carbon flux tower sites used for predicting annual gross primary productivity (GPP) over the global domain. The base map shows the distribution of biomes as determined from the MODIS MCD12Q1-Type2 (UMD) ^80^ global land cover classification. The figure was created in the ArcMap software environment ^81^.


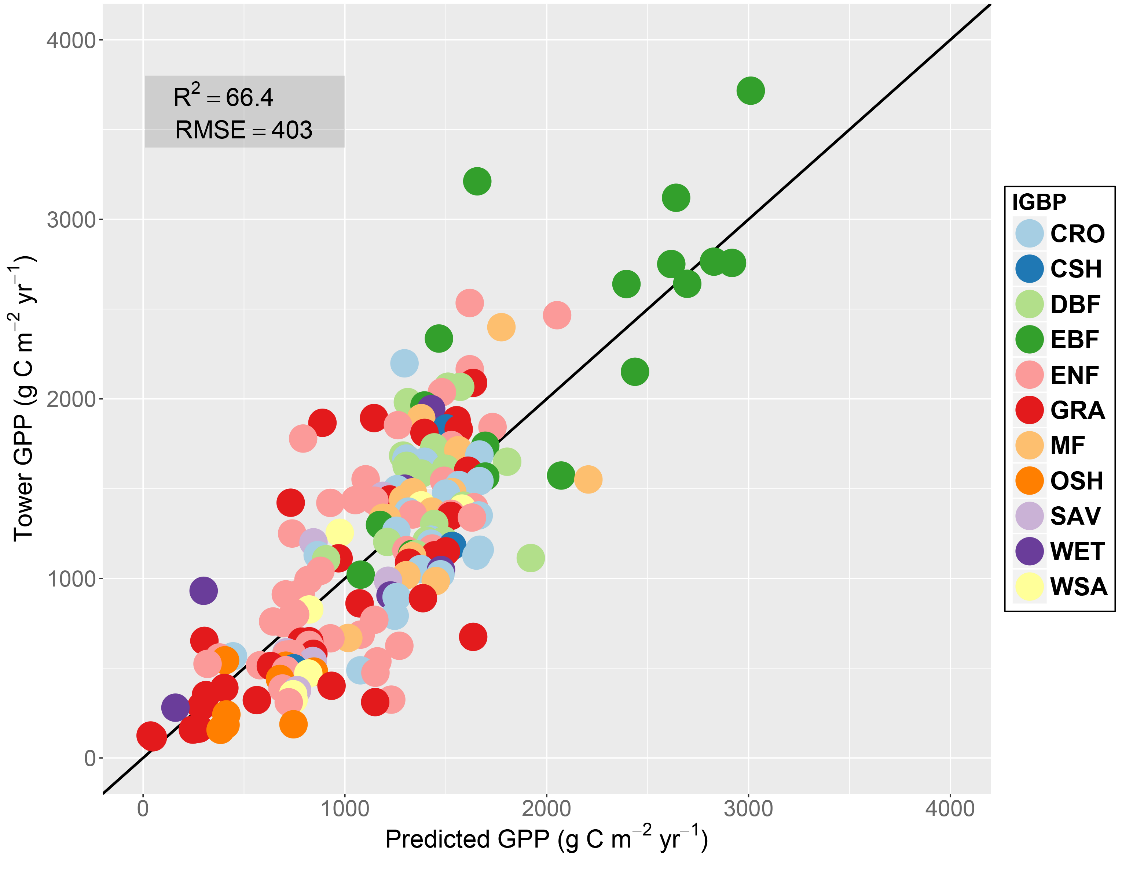


**Figure S7.** Comparison of annual GPP (g C m^-2^ yr^-1^) estimated from the trait-based GAM relative to tower GPP observations from all 164 global tower sites. The dominant plant functional type of each tower site indicated from the collocated MODIS MCD12Q1 global land cover map^80^ is depicted by the different colors.


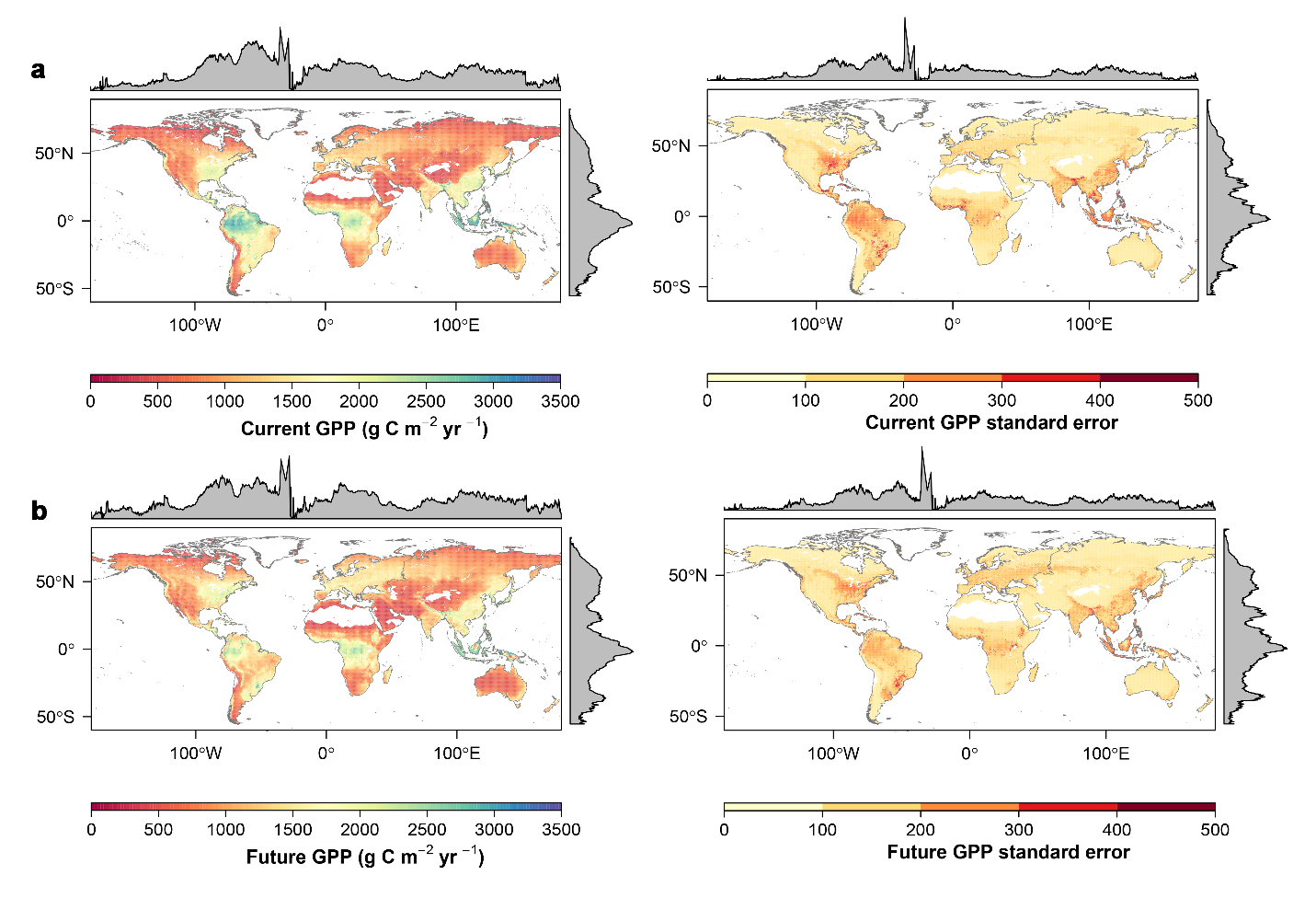


**Figure S8.** Global distribution of estimated maximum annual GPP for current (a) and projected future (b) climate conditions based on key plant traits and associated standard error maps of the model estimates (g C m^-2^ yr^-1^). Grey shaded plots along the X- and Y-axes denote relative variations in GPP spatial means for 0.05° latitude and longitude increments. The figure was created using the rasterVis library ^82^ in R ^83^.


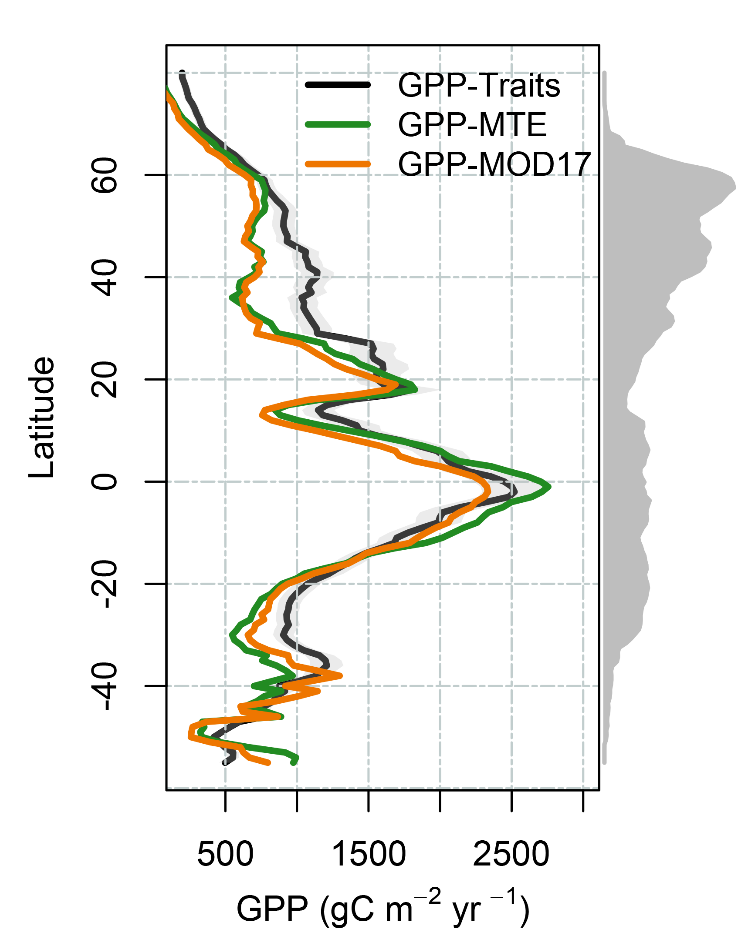


**Figure S9.** Latitudinal averages of estimated annual gross primary productivity derived from this study (GPP-Traits) in relation to other available GPP data records from MODIS (GPP-MOD17)^84^ and MPI tower observation up-scaled GPP (GPP-MTE)^85^. Gray shading on the secondary Y axis shows the relative land area represented within each 0.05° latitudinal bin. Similar grey shading in the plot represents the spatial standard deviation of the GPP-Traits results within each latitudinal bin. In this study, current GPP is estimated from global plant traits under current climate conditions determined using observed annual GPP accumulations from tower carbon flux sites representing the major global biomes. Barren and sparsely vegetated areas are masked out in order to provide consistent estimates across the datasets.


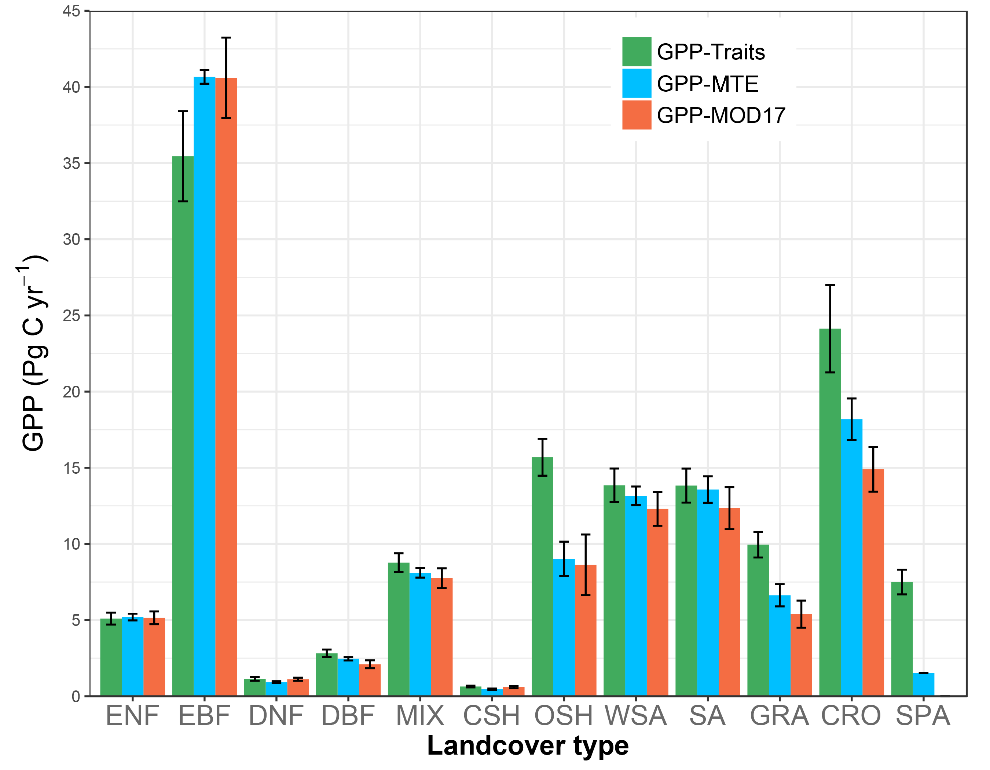


**Figure S10.** Comparison between current GPP estimates based on traits data from this study (GPP-Traits) in relation to average annual GPP from MODIS (GPP-MOD17) and MPI (GPP-MTE) records for different global biome types defined from the MODIS land cover map ^80^ including: ENF (Evergreen Needle leaf Forest); EBF ( Evergreen Broadleaf Forest); DNF (Deciduous Needle leaf Forest); DBF (Deciduous Broadleaf Forest); MF (Mixed Forest); CSH (Closed Shrubland); OSH (Open Shrubland); WSA (Woody Savanna); SA (Savanna); GRA (Grassland); CRO (Croplands); and SPA (Sparse Vegetation, areas with annual precipitation of less than 50mm are masked out). Error bars denote the standard error of the estimated current GPP, and temporal standard deviations of the GPP records from MOD17 and MTE.


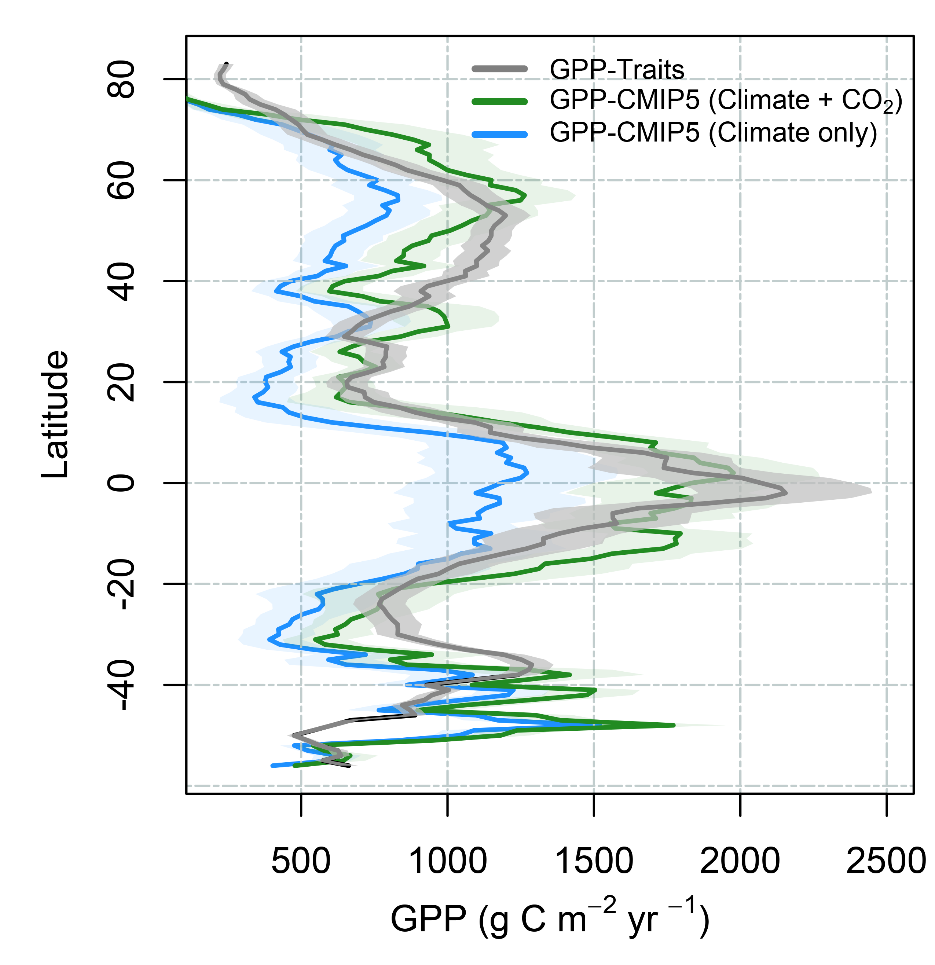


**Figure S11.** **Mean latitudinal distributions of alternative GPP estimates under projected future climate conditions.**  Comparison between projected future GPP from this study and the ensemble mean GPP from five CMIP5 models with productivity estimates for the year 2070 with and without CO_2_ fertilization effect on GPP. Shaded colors represent standard deviations of the range of model estimates.


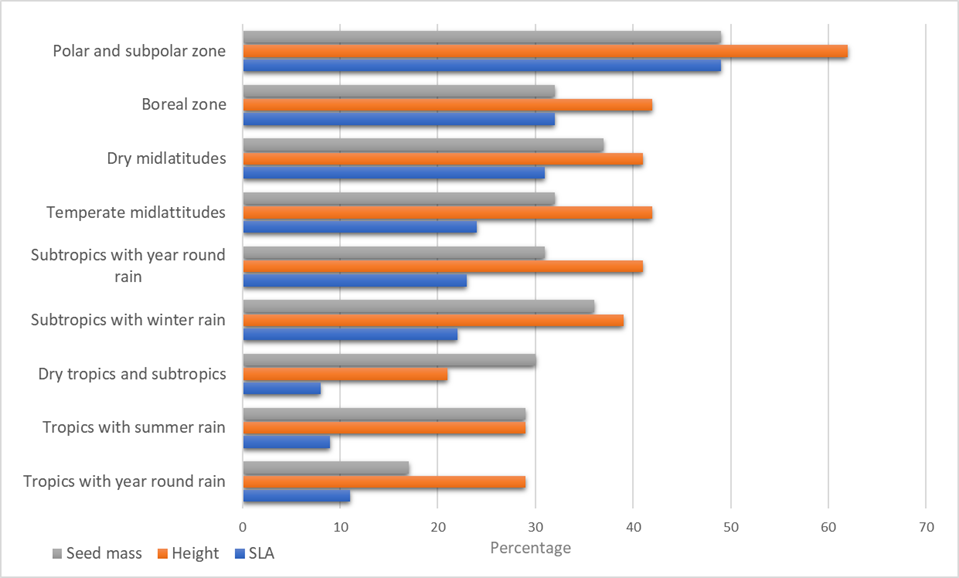


**Figure S12.** **Percentage of species trait data in the TRY database matching with the number of species reported in the sPlot database** ^86^ **for the major global biomes.**

**Supplementary References**

1. Atkin, O., Westbeek, M., Cambridge, M. L., Lambers, H. & Pons, T. L. Leaf respiration in light and darkness (a comparison of slow-and fast-growing Poa species). *Plant Physiol.* **113,** 961–965 (1997).

2. Campbell, C. *et al.* Acclimation of photosynthesis and respiration is asynchronous in response to changes in temperature regardless of plant functional group. *New Phytol.* **176,** 375–389 (2007).

3. Atkin, O. K., Schortemeyer, M., McFarlane, N. & Evans, J. R. The response of fast- and slow-growing Acacia species to elevated atmospheric CO2: an analysis of the underlying components of relative growth rate. *Oecologia* **120,** 544–554 (1999).

4. Loveys, B. . *et al.* Thermal acclimation of leaf and root respiration: an investigation comparing inherently fast‐and slow‐growing plant species. *Glob. Chang. Biol.* **9,** 895–910 (2003).

5. Xu, L. & Baldocchi, D. D. Seasonal trends in photosynthetic parameters and stomatal conductance of blue oak (Quercus douglasii) under prolonged summer drought and high temperature. *Tree Physiol.* **23,** 865–77 (2003).

6. Cavender-Bares, J., Keen, A. & Miles, B. Phylogenetic Structure of Floridian Plant Communities Depends on Taxonomic and Spatial Scale. *Ecology* **87,** S109–S122 (2006).

7. Quested, H. M. *et al.* Decomposition of sub-arctic plants with differing nitrogen economies: a functional role for hemiparasites. *Ecol.* **84,** 3209–3221 (2003).

8. Cornelissen, J. H. C. *et al.* Leaf digestibility and litter decomposability are related in a wide range of subarctic plant species and types. *Funct. Ecol.* **18,** 779–786 (2004).

9. Cornelissen, J., Diez, P. & Hunt, R. Seedling growth, allocation and leaf attributes in a wide range of woody plant species and types. *J. Ecol.* **84,** 755–765 (1996).

10. Díaz, S. *et al.* The plant traits that drive ecosystems: Evidence from three continents. *J. Veg. Sci.* **15,** 295 (2004).

11. Cornelissen, J. An experimental comparison of leaf decomposition rates in a wide range of temperate plant species and types. *J. Ecol.* **84,** 573–582 (1996).

12. Cornelissen, J. H. C. H. C. *et al.* Functional traits of woody plants: correspondence of species rankings between field adults and laboratory‐grown seedlings? *J. …* **14,** 311–322 (2003).

13. Preston, K., Cornwell, W. & DeNoyer, J. Wood density and vessel traits as distinct correlates of ecological strategy in 51 California coast range angiosperms. *New Phytol.* **170,** 807–18 (2006).

14. Han, W., Fang, J., Guo, D. & Zhang, Y. Leaf nitrogen and phosphorus stoichiometry across 753 terrestrial plant species in China. *New Phytol.* **168,** 377–385 (2005).

15. Freschet, G. T., Cornelissen, J. H. C., van Logtestijn, R. S. P. & Aerts, R. Evidence of the ‘plant economics spectrum’ in a subarctic flora. *J. Ecol.* **98,** 362–373 (2010).

16. Garnier, E. *et al.* Assessing the effects of land-use change on plant traits, communities and ecosystem functioning in grasslands: a standardized methodology and lessons from an application to 11 European sites. *Ann. Bot.* **99,** 967–85 (2007).

17. Hickler, T. Plant functional types and community characteristics along environmental gradients on Öland’s Great Alvar (Sweden). (Lund, 1999).

18. Kattge, J., Knorr, W., Raddatz, T. & Wirth, C. Quantifying photosynthetic capacity and its relationship to leaf nitrogen content for global-scale terrestrial biosphere models. *Glob. Chang. Biol.* **15,** 976–991 (2009).

19. Kleyer, M. *et al.* The LEDA Traitbase: a database of life-history traits of the Northwest European flora. *J. Ecol.* **96,** 1266–1274 (2008).

20. Laughlin, D. C., Leppert, J. J., Moore, M. M. & Sieg, C. H. A multi-trait test of the leaf-height-seed plant strategy scheme with 133 species from a pine forest flora. *Funct. Ecol.* **24,** 493–501 (2010).

21. Baker, T. R. *et al.* Basin-wide variations in foliar properties of Amazonian forest: phylogeny, soils and climate. *Biogeosciences* **6,** 2677–2708 (2009).

22. Medlyn, B. E. *et al.* Effects of elevated [CO2] on photosynthesis in European forest species: a meta‐analysis of model parameters. *Plant. Cell Environ.* **22,** 1475–1495 (1999).

23. Messier, J., McGill, B. J. & Lechowicz, M. J. How do traits vary across ecological scales? A case for trait-based ecology. *Ecol. Lett.* **13,** 838–48 (2010).

24. Niinemets, Ü. Global-scale climatic controls of leaf dry mass per area, density, and thickness in trees and shrubs. *Ecology* **82,** 453–469 (2001).

25. Onoda, Y. *et al.* Global patterns of leaf mechanical properties. *Ecol. Lett.* **14,** 301–12 (2011).

26. Ordoñez, A. J. C. *et al.* Leaf habit and woodiness regulate different leaf economy traits at a given nutrient supply. *Ecology* **91,** 3218–3228 (2010).

27. Ogaya, R. & Peñuelas, J. Comparative field study of Quercus ilex and Phillyrea latifolia: photosynthetic response to experimental drought conditions. *Environ. Exp. Bot.* **50,** 137–148 (2003).

28. Penuelas, J. *et al.* Faster returns on ‘leaf economics’ and different biogeochemical niche in invasive compared with native plant species. *Glob. Chang. Biol.* **16,** 2171–2185 (2009).

29. Poorter, L. Leaf traits show different relationships with shade tolerance in moist versus dry tropical forests. *New Phytol.* **181,** 890–900 (2009).

30. Reich, P. B., Oleksyn, J. & Wright, I. J. Leaf phosphorus influences the photosynthesis-nitrogen relation: a cross-biome analysis of 314 species. *Oecologia* **160,** 207–12 (2009).

31. Reich, P. B. *et al.* Scaling of respiration to nitrogen in leaves, stems and roots of higher land plants. *Ecol. Lett.* **11,** 793–801 (2008).

32. Shipley, B. & Vu, T.-T. Dry matter content as a measure of dry matter concentration in plants and their parts. *New Phytol.* **153,** 359–364 (2002).

33. Shipley, B. Trade-offs between net assimilation rate and specific leaf area in determining relative growth rate: relationship with daily irradiance. *Funct. Ecol.* **16,** 682–689 (2002).

34. Shipley, B. & Lechowicz, M. The functional co-ordination of leaf morphology, nitrogen concentration, and gas exchange in 40 wetland species. *Ecoscience* **7,** 183–194 (2000).

35. Pyankov, V., Kondratchuk, A. & Shipley, B. Leaf structure and specific leaf mass: the alpine desert plants of the Eastern Pamirs, Tadjikistan. *New Phytol.* **143,** 131–142 (1999).

36. Meziane, D. & Shipley, B. Interacting components of interspecific relative growth rate: constancy and change under differing conditions of light and nutrient supply. *Funct. Ecol.* **13,** 611–622 (1999).

37. Shipley, B. Structured interspecific determinants of specific leaf area in 34 species of herbaceous angiosperms. *Funct. Ecol.* **9,** 312–319 (1995).

38. van Bodegom, P. M., Sorrell, B. K., Oosthoek, A., Bakker, C. & Aerts, R. Separating the effects of partial submergence and soil oxygen demand on plant physiology. *Ecology* **89,** 193–204 (2008).

39. Wright, I. J. *et al.* The worldwide leaf economics spectrum. *Nature* **428,** 821–7 (2004).

40. Wright, I. J. *et al.* Relationships among ecologically important dimensions of plant trait variation in seven neotropical forests. *Ann. Bot.* **99,** 1003–15 (2007).

41. Fonseca, C. R., Overton, J. M., Collins, B. & Westoby, M. Shifts in trait-combinations along rainfall and phosphorus gradients. *J. Ecol.* **88,** 964–977 (2000).

42. Craine, J. M., Lee, W. G., Bond, W. J., Williams, R. J. & Johnson, L. C. Environmental constraints on a global relationship among leaf and root traits of grasses. *Ecology* **86,** 12–19 (2005).

43. Craine, J. M. *et al.* Global patterns of foliar nitrogen isotopes and their relationships with climate, mycorrhizal fungi, foliar nutrient concentrations, and nitrogen availability. *New Phytol.* **183,** 980–992 (2009).

44. Kazakou, E., Vile, D., Shipley, B., Gallet, C. & Garnier, E. Co-variations in litter decomposition, leaf traits and plant growth in species from a Mediterranean old-field succession. *Funct. Ecol.* **20,** 21–30 (2006).

45. Gavin, D. D. G. *et al.* Climate refugia: joint inference from fossil records, species distribution models and phylogeography. *New Phytol.* **185,** 19–28 (2014).

46. Bakker, C., Rodenburg, J. & van Bodegom, P. M. Effects of Ca- and Fe-rich Seepage on P Availability and Plant Performance in Calcareous Dune Soils. *Plant Soil* **275,** 111–122 (2005).

47. Bi, D. *et al.* The ACCESS coupled model : description , control climate and evaluation. *Aust. Meteorol. Oceanogr. J.* **63,** 41–64 (2013).

48. Wu, T. *et al.* Global carbon budgets simulated by the Beijing Climate Center Climate System Model for the last century. *J. Geophys. Res. Atmos.* **118,** 4326–4347 (2013).

49. Gent, P. R. *et al.* The community climate system model version 4. *J. Clim.* **24,** 4973–4991 (2011).

50. Voldoire, a. *et al.* The CNRM-CM5.1 global climate model: Description and basic evaluation. *Clim. Dyn.* **40,** 2091–2121 (2013).

51. Donner, L. J. *et al.* The dynamical core, physical parameterizations, and basic simulation characteristics of the atmospheric component AM3 of the GFDL global coupled model CM3. *J. Clim.* **24,** 3484–3519 (2011).

52. Schmidt, G. a *et al.* Configuration and assessment of the GISS ModelE2 contributions to the CMIP5 archive. *J. Adv. Model. Earth Syst.* **6,** 141–184 (2014).

53. Martin, G. M. *et al.* The HadGEM2 family of Met Office Unified Model climate configurations. *Geosci. Model Dev.* **4,** 723–757 (2011).

54. Volodin, E. M., Dianskii, N. a. & Gusev, a. V. Simulating present-day climate with the INMCM4.0 coupled model of the atmospheric and oceanic general circulations. *Izv. Atmos. Ocean. Phys.* **46,** 414–431 (2010).

55. Dufresne, J. L. *et al.* *Climate change projections using the IPSL-CM5 Earth System Model: From CMIP3 to CMIP5*. *Climate Dynamics* **40,** (2013).

56. Watanabe, S. *et al.* MIROC-ESM: model description and basic results of CMIP5-20c3m experiments. *Geosci. Model Dev. Discuss.* **4,** 1063–1128 (2011).

57. Watanabe, M. *et al.* Improved climate simulation by MIROC5: Mean states, variability, and climate sensitivity. *J. Clim.* **23,** 6312–6335 (2010).

58. Block, K. & Mauritsen, T. Forcing and feedback in the MPI-ESM-LR coupled model under abruptly quadrupled CO 2. *J. Adv. Model. Earth Syst.* **5,** 676–691 (2013).

59. Yukimoto, S. *et al.* A New Global Climate Model of the Meteorological Research Institute: MRI-CGCM3-Model Description and Basic Performance. *J. Meteorol. Soc. Japan* **90A,** 23–64 (2012).

60. Bentsen, M. *et al.* The Norwegian Earth System Model, NorESM1-M – Part 1: Description and basic evaluation. *Geosci. Model Dev. Discuss.* **5,** 2843–2931 (2012).

61. Hengl, T. *et al.* *SoilGrids250m: Global gridded soil information based on machine learning*. *Plos One* **12,** (2017).

62. Stoy, P. C., Trowbridge, A. M. & Bauerle, W. L. Controls on seasonal patterns of maximum ecosystem carbon uptake and canopy-scale photosynthetic light response: contributions from both temperature and photoperiod. *Photosynth. Res.* **119,** 49–64 (2014).

63. Sulkava, M., Luyssaert, S., Zaehle, S. & Papale, D. Assessing and improving the representativeness of monitoring networks: The European flux tower network example. *J. Geophys. Res.* **116,** G00J04 (2011).

64. Bonan, G. B. *et al.* Improving canopy processes in the Community Land Model version 4 (CLM4) using global flux fields empirically inferred from FLUXNET data. *J. Geophys. Res.* **116,** G02014 (2011).

65. Schwalm, C. R. *et al.* Assimilation exceeds respiration sensitivity to drought: A FLUXNET synthesis. *Glob. Chang. Biol.* **16,** 657–670 (2010).

66. Blyth, E. *et al.* A comprehensive set of benchmark tests for a land surface model of simultaneous fluxes of water and carbon at both the global and seasonal scale. *Geosci. Model Dev.* **4,** 255–269 (2011).

67. Hilton, T. W., Davis, K. J., Keller, K. & Urban, N. M. Improving North American terrestrial CO2 flux diagnosis using spatial structure in land surface model residuals. *Biogeosciences* **10,** 4607–4625 (2013).

68. Piao, S. *et al.* Net carbon dioxide losses of northern ecosystems in response to autumn warming. *Nature* **451,** 49–52 (2008).

69. Imer, D., Merbold, L., Eugster, W. & Buchmann, N. Temporal and spatial variations of soil CO2, CH4 and N2O fluxes at three differently managed grasslands. *Biogeosciences* **10,** 5931–5945 (2013).

70. Division, E. S. *et al.* On the separation of net ecosystem exchange into assimilation and ecosystem respiration: review and improved algorithm. *Glob. Chang. Biol.* **11,** 1424–1439 (2005).

71. Marek, M. V. *et al.* Carbon exchange between ecosystems and atmosphere in the Czech Republic is affected by climate factors. *Environ. Pollut.* **159,** 1035–1039 (2011).

72. Gilmanov, T. G. *et al.* Productivity, Respiration, and Light-Response Parameters of World Grassland and Agroecosystems Derived From Flux-Tower Measurements. *Rangel. Ecol. Manag.* **63,** 16–39 (2010).

73. Wohlfahrt, G. *et al.* Biotic, abiotic, and management controls on the net ecosystem CO 2 exchange of European mountain grassland ecosystems. *Ecosystems* **11,** 1338–1351 (2008).

74. Horn, J. E. & Schulz, K. Identification of a general light use efficiency model for gross primary production. *Biogeosciences* **8,** 999–1021 (2011).

75. Hilton, T. W., Davis, K. J. & Keller, K. Evaluating terrestrial CO2 flux diagnoses and uncertainties from a simple land surface model and its residuals. *Biogeosciences* **11,** 217–235 (2014).

76. Barron-Gafford, G. a., Scott, R. L., Jenerette, G. D. & Huxman, T. E. The relative controls of temperature, soil moisture, and plant functional group on soil CO 2 efflux at diel, seasonal, and annual scales. *J. Geophys. Res.* **116,** G01023 (2011).

77. Lasslop, G. *et al.* Separation of net ecosystem exchange into assimilation and respiration using a light response curve approach: critical issues and global evaluation. *Glob. Chang. Biol.* **16,** 187–208 (2010).

78. Yi, C. *et al.* Climate control of terrestrial carbon exchange across biomes and continents. *Environ. Res. Lett.* **5,** 34007 (2010).

79. Ciais, P. *et al.* The carbon balance of Africa: synthesis of recent research studies. *Philos. Trans. R. Soc. A Math. Phys. Eng. Sci.* **369,** 2038–2057 (2011).

80. Friedl, M. a. *et al.* MODIS Collection 5 global land cover: Algorithm refinements and characterization of new datasets. *Remote Sens. Environ.* **114,** 168–182 (2010).

81. ESRI. ArcGIS Desktop: Release 10. (2011).

82. Hijmans, R. & Lamigueiro, O. P. meteoForecast. (2016). R package version 0.43.

83. R Core Team. R: A language and environment for statistical computing. (2016).

84. Zhao, M. & Running, S. W. Drought-induced reduction in global terrestrial net primary production from 2000 through 2009. *Science* **329,** 940–3 (2010).

85. Jung, M. *et al.* Global patterns of land-atmosphere fluxes of carbon dioxide, latent heat, and sensible heat derived from eddy covariance, satellite, and meteorological observations. *J. Geophys. Res.* **116,** 1–16 (2011).

86. Dengler, J. *et al.* sPlot - the new global vegetation-plot database for addressing traitenvironment relationships across the world’s biomes. *Biodivers. Veg. pattern, Process. Conserv.* 90 (2014). doi:10.13140/RG.2.1.1979.0164
